# Reprogramming of Gene Transcripts and Metabolites by the Wild Soybean Endophyte *Pseudomonas* sp. 77S3 Improves Soybean Salt Tolerance

**DOI:** 10.1111/pbi.70514

**Published:** 2025-12-29

**Authors:** Wanying Zhang, Chengyang Song, Tianqi Wang, Xiulin Liu, Yisheng Fang, Zhu Yan, Yaxi Zhu, Na Zheng, Xiaofei Ma, Guochen Qin, Dan Zhu, Junchuan Xiao, Xing Wang Deng, Xiao Luo

**Affiliations:** ^1^ Shandong Key Laboratory of Precision Molecular Crop Design and Breeding, Peking University Institute of Advanced Agricultural Sciences Shandong Laboratory of Advanced Agricultural Sciences in Weifang Shandong China; ^2^ School of Advanced Agricultural Sciences Peking University Beijing China; ^3^ College of Life Science, Key Laboratory of Plant Biotechnology in Universities of Shandong Province Qingdao Agricultural University Qingdao China; ^4^ Shenzhen Weizhi Biotech Co. Limited Shenzhen China

**Keywords:** endophyte, metabolic, *Pseudomonas*, reprogramming, salt, soybean

## Abstract

Soybean is a critical source of protein and vegetable oil worldwide. Expanding its cultivation into salinity lands represents a promising strategy for increasing production; however, soil salinity severely limits soybean growth by disrupting physiological and metabolic homeostasis. Although beneficial endophytes can enhance plant stress adaptation, the molecular mechanisms by which they reprogram host responses under salinity remain poorly understood. In this study, we isolated *Pseudomonas* sp. 77S3 from salt‐tolerant wild soybean and demonstrated its exceptional ability to significantly improve growth and salt tolerance in cultivated soybean under salt stress, using both fresh and fermented formulations. Integrated transcriptomic and metabolomic analyses revealed that 77S3 inoculation systemically reprograms gene expression and metabolic networks in soybean roots. Key to this reprogramming was the enhancement of nitrogen metabolism, orchestrated largely by the nitrate transporter NRT1.5, which facilitated nitrogen reallocation under stress. Functional studies using *nrt1.5* knockdown lines confirmed that NRT1.5 is essential for 77S3‐mediated improvements in salt tolerance, ion homeostasis, root architecture remodelling, and carbon–nitrogen rebalancing. Additionally, 77S3 increased antioxidant capacity, modulated phytohormone signalling, particularly in auxin and ethylene pathways, and improved phosphorus and potassium solubilisation. These multi‐level adaptations collectively enhance salinity resilience in soybean. Our findings provide novel insights into the mechanistic basis of endophyte‐induced salt tolerance and support the use of *Pseudomonas* sp. 77S3 as a sustainable bioinoculant for soybean production in saline agriculture.

## 1 Introduction

Soil salinity is a critical constraint in agricultural productivity, adversely affecting crop growth by impairing soil structure, reducing water and air permeability, altering osmotic balance, and disrupting ion homeostasis (Busoms et al. 2023). Soybean ( *Glycine max* ), a major legume crop, is highly susceptible to salt stress, which inhibits germination, reduces photosynthetic efficiency, and compromises overall plant development, leading to significant yield and quality losses. Conventional approaches to address soil salinity, such as excessive irrigation or chemical fertilisation, are often unsustainable. Over‐irrigation can exacerbate root diseases and water waste, while heavy fertiliser use contributes to environmental pollution and ecosystem degradation. Thus, there is an urgent need to develop eco‐friendly and efficient strategies to enhance soybean adaptation to saline environments.

Endophytic microorganisms, which colonise internal plant tissues without causing disease, have emerged as promising agents for improving plant stress resistance and nutrient acquisition (Farrar et al. 2014). Unlike epiphytes, which reside on plant surfaces, endophytes intimately interact with host tissues and modulate physiological processes critical for growth and stress adaptation (Gu et al. 2024; Han et al. 2020; Zhang et al. 2020). They contribute to plant health through biological nitrogen fixation (Sun et al. 2022), phytohormone synthesis such as auxin (Gonzalez Ortega‐Villaizan et al. 2024; Han et al. 2020), siderophore production (Li et al. 2008; Lin et al. 2021), and solubilisation of phosphorus and potassium (de Almeida Lopes et al. 2016), thereby facilitating nutrient acquisition and providing plants with a robust defence mechanism against stress conditions. These attributes make endophytes valuable tools for sustainable agriculture, particularly under abiotic stresses such as salinity.

Modern cultivated soybean varieties, resulting from intensive breeding, often exhibit reduced genetic diversity and compromised stress adaptability. In contrast, wild soybean ( *Glycine soja* ) relatives possess rich genetic resources and host diverse endophytic communities, which may contribute to their resilience under adverse conditions. However, habitat loss and agricultural expansion threaten wild soybean populations, risking the erosion of beneficial microbial associations. Reintroducing endophytes from wild soybeans into cultivated varieties offers a promising approach to restore stress tolerance traits and reduce dependency on chemical inputs (Lata et al. 2018). Although some members of the genus *Pseudomonas* (hereinafter referred to as *Pseudomonas* sp.) have been reported to exhibit biocontrol and plant growth‐promoting (PGP) effects in various crops (Andelkovic et al. 2025), their mechanisms of action in soybean, particularly under salt stress, remain poorly understood. Moreover, the molecular mechanisms underlying endophyte‐mediated salt tolerance have not been fully elucidated.

Nitrogen (N) is fundamental for soybean growth, development, and ultimate yield formation. However, the efficient acquisition and utilisation are often impaired under salt stress. Nitrate Transporter 1/Peptide Transporter (NRT1/PTR) family (NPF) proteins play pivotal roles in nitrogen allocation and assimilation, contributing not only to nutrient homeostasis but also to adaptive responses under abiotic stresses such as salinity (Wu et al. 2025; Zhou et al. 2025). In Arabidopsis, *NRT1.5* mediates nitrate loading into the xylem and facilitates its root‐to‐shoot translocation. Knockdown or knockout *NRT1.5* exhibits impaired long‐distance nitrate transport and is compromised in stress‐induced nitrogen reallocation, underscoring its critical role in adaptive responses to abiotic stress (Lin et al. 2008; Meng et al. 2016). However, the functional significance of *NRT1.5* homologues in soybean remains poorly characterised. Moreover, while beneficial plant–microbe interactions are known to enhance nutrient use efficiency, the potential for endophytic bacteria to modulate soybean salt tolerance by reprogramming nitrogen metabolism, potentially through key transporters like *NRT1.5* to facilitate nitrogen reallocation and assimilation under stress, has not been investigated.

Recent advances in multi‐omics technologies provide unprecedented opportunities to decipher plant–microbe interactions at molecular and metabolic levels. In this study, we isolated a salt‐tolerant endophyte, *Pseudomonas* sp. 77S3, from wild soybean and demonstrated its efficacy in promoting growth and salt tolerance in cultivated soybean. We employed transcriptomic and metabolomic analyses to investigate the mechanistic basis of 77S3‐induced resilience under saline conditions. Our results show that inoculation with 77S3 reprograms nitrogen metabolism, primarily through *NRT1.5*‐dependent metabolic remodelling, while also enhancing antioxidant capacity and modulating phytohormone signalling. These synergistic effects collectively improve soybean adaptation to salt stress. These insights not only highlight the potential of 77S3 as a bioinoculant but also contribute to a broader understanding of endophyte‐mediated stress resilience in crops.

## 2 Results

### 2.1 Isolation and Characterisation of the Salt‐Tolerant Endophyte *Pseudomonas* sp. 77S3

Endophytic microorganisms were isolated from the roots, stems, and leaves of salt‐tolerant wild soybean plants. The endophytic origin of these isolates was confirmed by the absence of microbial growth in the final wash step. Among the 80 distinct strains obtained, *Pseudomonas* represented the dominant genus, comprising 15 isolates (Figure S1A). Salt tolerance screening on saline‐alkaline medium showed that strains from wild soybean accessions 77 and 41 exhibited superior growth under stress (Table S2). Whole‐plant salt tolerance assays further confirmed that accession 77 possessed stronger salt tolerance than accession 41 (Figure S1B). Accordingly, ten salt‐tolerant strains isolated from accession 77 were selected for further study, including six from roots, three from stems, and one from leaf tissue (Figure S1C).

All isolates except 77R2 exhibited robust growth on nutrient agar (NA) medium supplemented with 500 mM NaCl at pH 9.0, demonstrating pronounced salt‐alkali tolerance (Figure S1C). Functional screening revealed that strain 77S3 exerted the most significant promotive effects on seed germination, plant growth, and salt tolerance (Figure S1D–F). Phenotypic and molecular characterisation revealed that strains 77R1 and 77S3 were identical; thus, 77S3 was chosen for further study.

**FIGURE 1:**
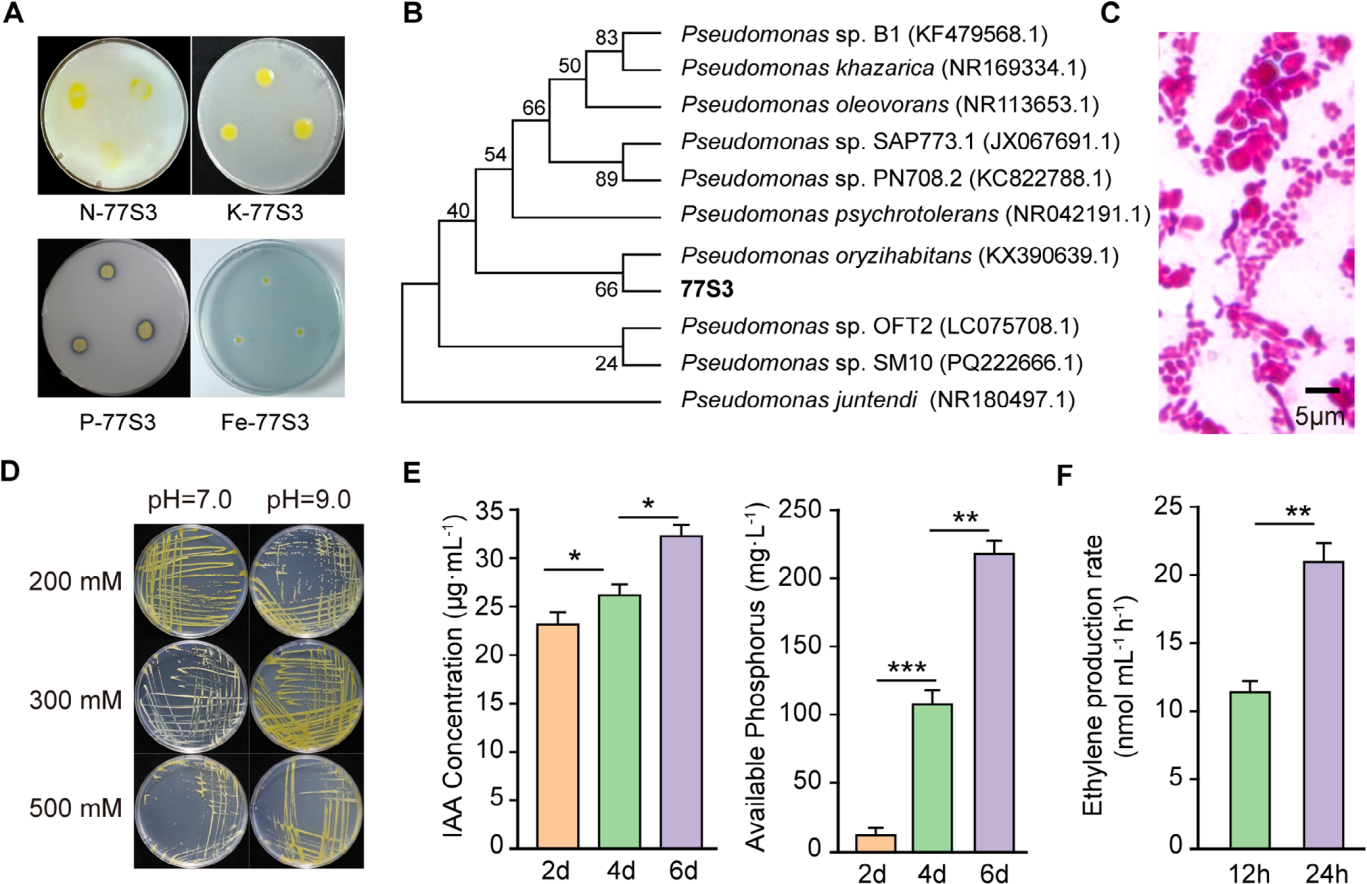
Isolation and characterisation of the salt‐resistant endophyte strain *Pseudomonas* sp. 77S3. (A) Functional characterisation of 77S3 grown on selective media: Nitrogen fixation (Burk's medium), potassium solubilisation (potassium bacteria agar), phosphate solubilisation (NBRIP medium), and siderophore production (chrome azurol‐S agar). (B) Neighbour‐joining phylogenetic tree constructed from 16S rRNA gene sequences, indicating the taxonomic position of strain 77S3 within related *Pseudomonas* species. (C) Gram staining of strain 77S3, confirming its gram‐negative rod‐shaped morphology. (D) Growth of strain 77S3 on nutrient agar under varying NaCl concentrations (200, 300, and 500 mM) and pH conditions (7.0 or 9.0). (E) Time‐course measurement of IAA production and soluble phosphorus content in the culture supernatant of 77S3 over 2, 4, and 6 days. (F) Ethylene production rates in 77S3 cultured in nitrogen‐free medium, measured at 12 and 24 h. (E and F) Data are presented as mean ± standard deviation (SD) (*n* = 5). Significant differences were determined by two‐tailed Student's *t*‐test (*p < 0.05, **p < 0.01, ***p < 0.001).

Colonies of 77S3 appeared yellow, round, moist, and shiny with raised centers and entire margins (Figure 1A). Initial phylogenetic analysis based on the 16S rRNA sequence identified strain 77S3 as *Pseudomonas* sp. (Figure 1B). For more precise taxonomic assignment, whole‐genome draft sequencing and multilocus sequence analysis (MLSA) of housekeeping genes (*dnaJ*, *dnaK*, *gyrA*, *gyrB*, *recA*, and *groEL*) were performed (Figure S2A,B). Phylogenetic classification via the GTDB‐Tk workflow indicated that strain 77S3 shares 98.33% average nucleotide identity (ANI) and an alignment fraction of 0.925 with the type strain of *Pseudomonas oryzihabitans* , confirming its species‐level identification (Figure S2A). Microscopic observation confirmed the Gram‐negative, rod‐shaped morphology of 77S3 (Figure 1C). The strain also demonstrated high salt tolerance, showing robust growth on NA medium amended with 500 mM NaCl at pH 9.0 (Figure 1D). Given its pronounced salt tolerance and growth‐promoting traits, *Pseudomonas* sp. 77S3 was selected for subsequent investigations into the molecular mechanisms mediating improved salt resilience in soybean.

### 2.2 Evaluation of Plant Growth‐Promoting Traits in *Pseudomonas* sp. 77S3

The PGP potential of 77S3 was evaluated using a series of biochemical assays, including tests for nitrogen fixation, phosphate and potassium solubilisation, siderophore production, and indoleacetic acid (IAA) synthesis. Plate‐based tests confirmed its ability to perform all these functions (Figure 1A,E). A clear solubilisation halo was observed around the colonies, with a phosphorus solubilisation ratio (D/d) of 1.68.

To quantify phosphate solubilisation ability, soluble phosphorus released by 77S3 was measured daily in a medium containing Ca_3_(PO_4_)_2_ as the sole phosphorus source, using the molybdenum antimony colorimetric method. The concentration of soluble phosphorus increased steadily over time, reaching 220 mg/L by the sixth day (Figure 1E). Similarly, IAA production in the bacterial culture was quantified colorimetrically, showing that 77S3 efficiently produced IAA, with secretion levels reaching 33.0 μg/mL after six days of incubation (Figure 1E).

To further verify nitrogen‐fixing capability, nitrogenase activity was assessed. The *nif*H gene, which encodes component II of nitrogenase and is widely used as a marker in studies of nitrogen‐fixing microbial communities (Zehr et al. 2003), was successfully amplified from 77S3 using specific primers, yielding a 286‐bp fragment (Figure S6A, Table S1). Nitrogenase activity was estimated via the ARA. Cultures incubated with 10% acetylene produced ethylene at detectable levels after 12 and 24 h, with rates of 11.51 ± 1.30 and 22.47 ± 2.62 nmol/mL/h, respectively, confirming the presence of functional nitrogenase in 77S3 (Figure 1F).

Comprehensive genomic analysis revealed that strain 77S3 harbours key functional genes supporting its multifaceted PGP potential. The genome encodes the complete *pqq* gene cluster (*pqqF*, *pqqB*, *pqqC*, *pqqD1*, and *pqqE*) responsible for phosphate solubilisation (Kim et al. 2003), *kup* for potassium uptake (Li et al. 2018), *Fer4_NifH* and *nifA* for nitrogen fixation, *nit1* and *trpC* for IAA biosynthesis, and *DHBA* for siderophore production (Porwal et al. 2015). Collectively, these results demonstrate that strain 77S3 possesses the ability for nitrogen fixation, phosphate and potassium solubilisation, siderophore synthesis, and auxin production, underscoring its potential as a PGP bacterium.

### 2.3 Root Colonisation by *Pseudomonas* sp. 77S3 Enhances Salt Tolerance Through Na^+^/K^+^ Homeostasis in Soybean Seedlings

**FIGURE 2:**
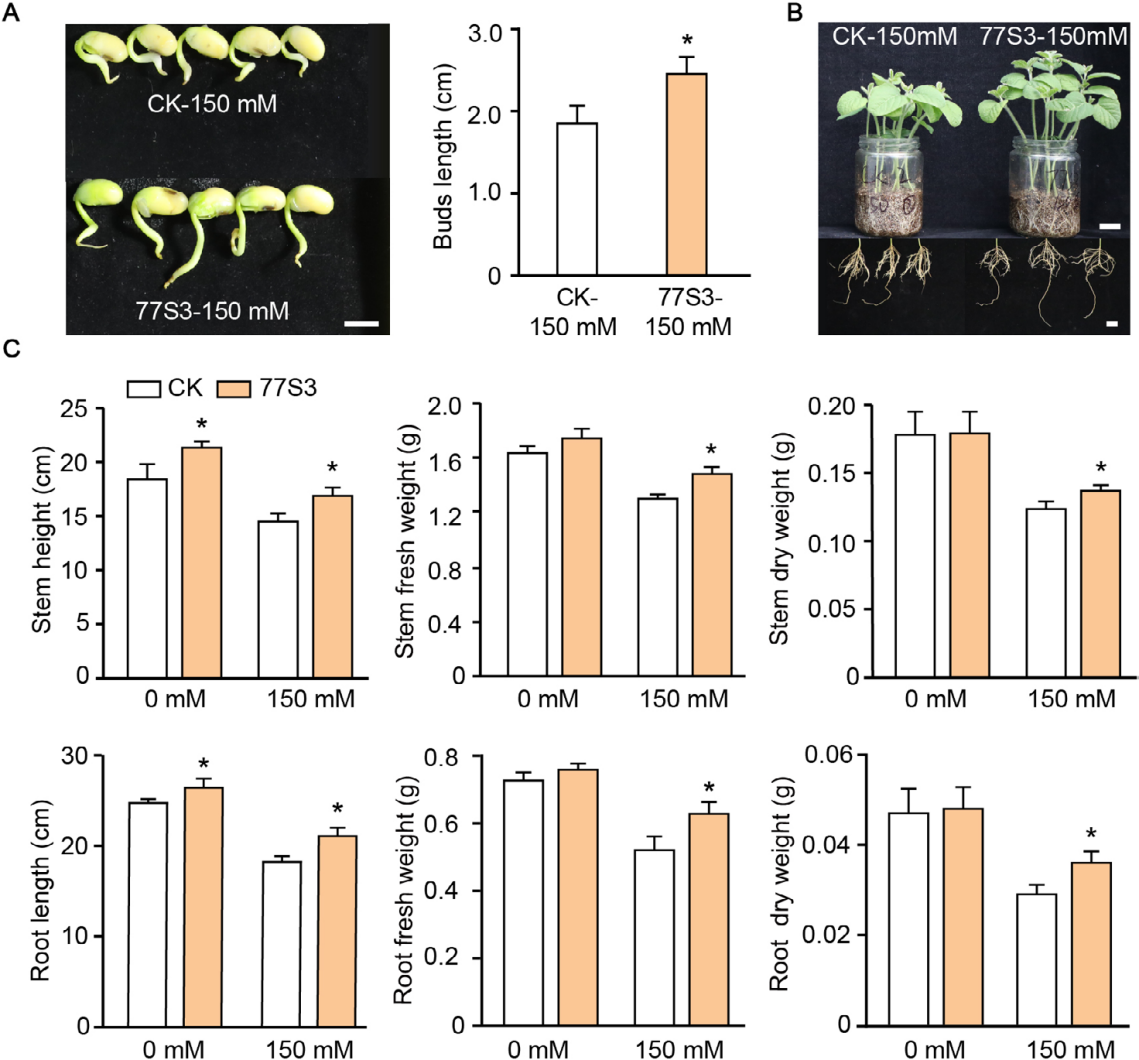
Effects of 77S3 inoculation on soybean growth under normal and salt stress conditions. (A) Phenotypic comparison of bud growth in control (CK) and 77S3 inoculated soybean lines under 150 mM NaCl stress, with the corresponding statistical analysis of bud length. Scale bar = 1 cm. (B) Phenotypic comparison of whole plants (including shoots and roots) between CK and 77S3 inoculated soybean lines under 150 mM salt stress. Scale bar = 2 cm. (C) Statistical analyses of stem height, root length, stem fresh weight, root fresh weight, stem dry weight, and root dry weight in CK and 77S3 inoculated soybean lines under 0 and 150 mM salt conditions. (A, C) Data are presented as mean ± SD (*n* = 5 and 10, respectively). Significant differences are indicated by * (*p* < 0.05, two‐tailed Student's *t*‐test).

Compared with the control group, treatment with the 77S3 suspension for five days increased the bud length by 26.2% (Figure 2A), indicating its strong potential to improve early‐stage growth under stress conditions. To further evaluate the growth‐promoting effect of 77S3 under salt stress, soybean seedlings were inoculated with 77S3 and subjected to 150 mM NaCl treatment. After 20 days, 77S3‐inoculated plants exhibited significantly improved salt tolerance and enhanced growth compared to uninoculated controls. Salt stress markedly inhibited growth in control plants, whereas 77S3‐inoculated seedlings showed reduced stress symptoms and greater vigour (Figure 2B). This was further supported by key growth parameters, with plant height increasing by 16.29%, root length by 15.73%, stem fresh weight and dry weight by 15.73% and 14.01%, respectively, and root fresh weight and dry weight by 20.77% and 24.14%, respectively (Figure 2C).

**FIGURE 3:**
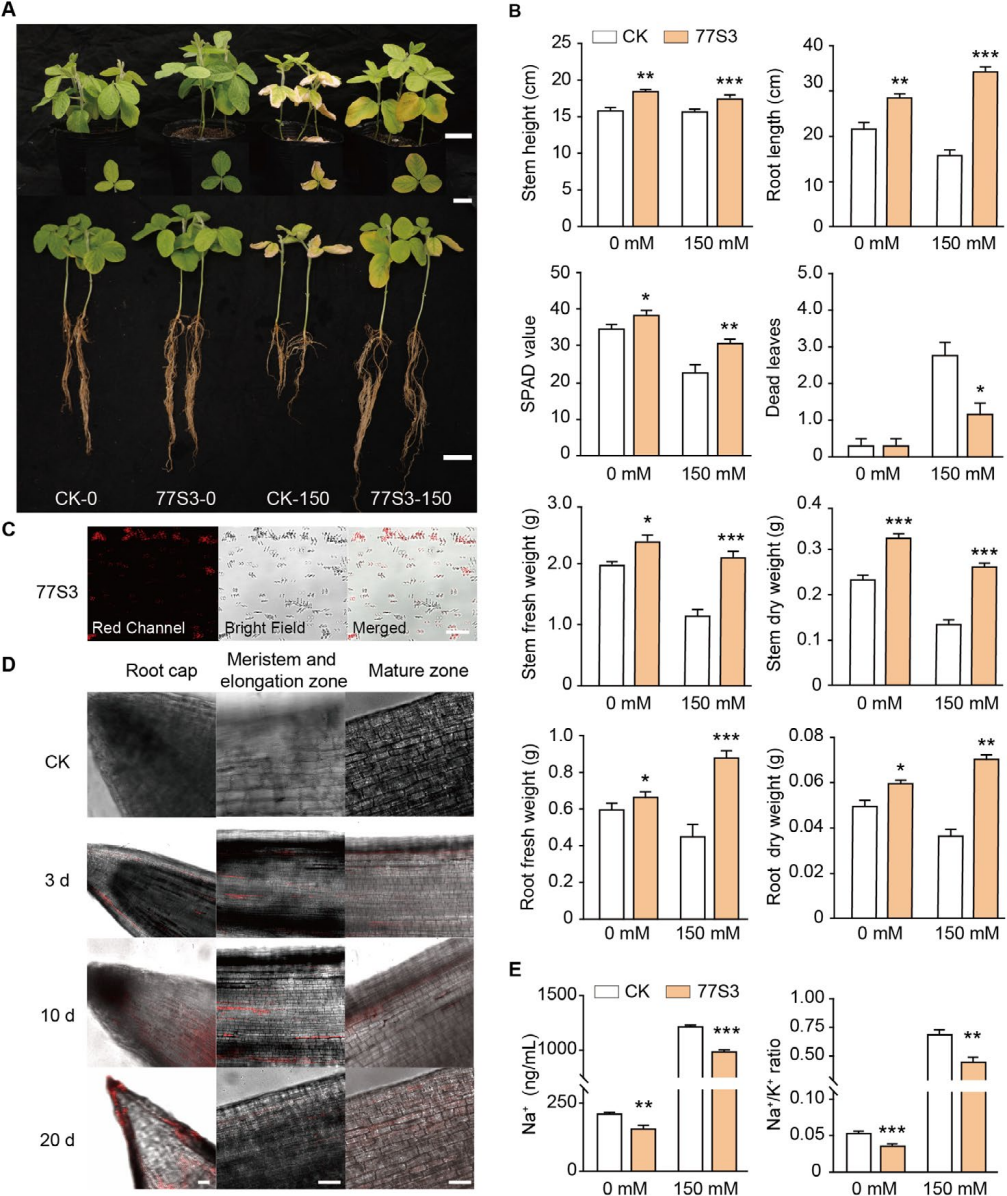
Effects of fermented 77S3 bacterial powder on soybean growth in soil under control and salt‐stress conditions. (A) Phenotypic response of soybean to 77S3 lyophilized inoculum under salt stress. Shoots (top), leaves (middle), and roots (bottom) of plants are shown following inoculation and exposure to 0 or 150 mM NaCl. Seven‐day‐old seedlings were inoculated with reconstituted bacterial suspension (OD_600_ = 1.0) prepared from lyophilized powder stored at 4°C for approximately one month. Viability assessment confirmed high activity of the resuscitated inoculum, with a viable cell concentration of approximately 2.3 × 10^10^ CFU/g. Salt treatment (150 mM NaCl) was initiated two days post‐inoculation, while control plants received sterile water throughout. Scale bar = 5 cm. (B) Quantitative measurements of stem height, root length, SPAD value, number of dead leaves, stem fresh/dry weight, and root fresh/dry weight in control (CK) and 77S3‐inoculated plants under 0 and 150 mM NaCl conditions. (C) RFP‐expressing 77S3 in pure culture imaged by confocal microscopy, showing red fluorescence (left), bright‐field (middle), and merged views (right). Scale bar = 20 μm. (D) Colonisation dynamics of soybean roots by RFP‐expressing 77S3 were examined using confocal microscopy. A selected confocal section from a Z‐stack with top and side orthogonal views at 3, 10, and 20 days post‐inoculation. Non‐inoculated roots (CK) imaged under identical settings show no fluorescence. Bar = 50 μm. (E) Determination of Na^+^ content and Na^+^/K^+^ ratio in CK and 77S3 inoculated soybean lines under 0 and 150 mM salt conditions. (B, E) Data are presented as mean ± SD (*n* = 10 and *n* = 3, respectively). Significant differences were determined by two‐tailed Student's *t*‐test (*p < 0.05, **p < 0.01, ***p < 0.001).

Considering that endophytic bacterial powder offers greater practicality as an inoculant than fresh cultures, *Pseudomonas* sp. 77S3 was developed into a lyophilized formulation through fermentation. After one month of storage at 4°C, the resuscitated inoculum exhibited high viability, with plate counts confirming a viable cell concentration of approximately 2.3 × 10^10^ CFU/g. Remarkably, the lyophilized powder retained substantial viability, exceeding 2.0 × 10^10^ CFU/g, even after six months of storage, demonstrating excellent long‐term stability and reliability for practical use as a microbial inoculant. Application of the bacterial powder to soil‐grown soybeans significantly promoted plant growth both with and without salt stress, increasing plant height, chlorophyll content, and biomass relative to controls (Figure 3A). Under normal conditions, inoculation increased chlorophyll content by 8.78%, plant height by 16.17%, and root length by 31.65%. Under salt stress, root length increased by 120.45%, chlorophyll content by 33.95%, and leaf abscission decreased by 57.14% (Figure 3B). These results demonstrate that 77S3 endophytic plays a crucial role in promoting soybean root development, photosynthesis, and overall plant health, thereby enhancing salt tolerance in soybean.

To investigate the colonisation dynamics of strain 77S3, soybean roots were sampled at 0 (non‐inoculated control), 3, 10, and 20 days post‐inoculation with an RFP‐tagged strain and examined by confocal laser scanning microscopy (CLSM). The RFP‐tagged strain displayed bright and specific fluorescence in axenic culture with negligible autofluorescence, confirming stable expression of the fluorescent label and clear detectability in plant tissues (Figure 3C). Comparative analysis with non‐inoculated controls revealed that 77S3‐RFP preferentially colonised the root meristematic and elongation zones, forming dense bacterial aggregates, while showing only sparse colonisation in the root crown and maturation zones (Figure 3D). Red fluorescence remained visible at 20 days, indicating sustained colonisation throughout the root system. Given the critical role of sodium (Na^+^) and potassium (K^+^) ion homeostasis in salt tolerance, ion concentrations were measured in inoculated and control roots. Interestingly, under both normal and salt‐stressed conditions, 77S3‐inoculated roots exhibited markedly reduced Na^+^ accumulation, resulting in a substantially lower Na^+^/K^+^ ratio compared to mock‐inoculated controls (Figure 3E). This optimised ion balance likely contributes to the maintenance of plant growth under salt stress.

### 2.4 Inoculation With 77S3 Reprograms the Soybean Root Transcriptome Under Salt Stress

To evaluate the transcriptional impact of 77S3 inoculation under salt stress, RNA‐seq was performed on soybean roots across inoculation and salinity conditions. All samples showed comparable read quality and alignment (Table S3). Of 52 872 transcripts mapped to the reference genome (Wm82.a4.v1), 37 192 (∼70%) were expressed (transcripts per million [TPM] > 1) in at least one treatment (Figure S3A, Table S4). DEGs were identified from the transcripts with valid expression using |log_2_(FC) ≥ 1| and FDR ≤ 0.05 (Tables S5 and S6).

**FIGURE 4:**
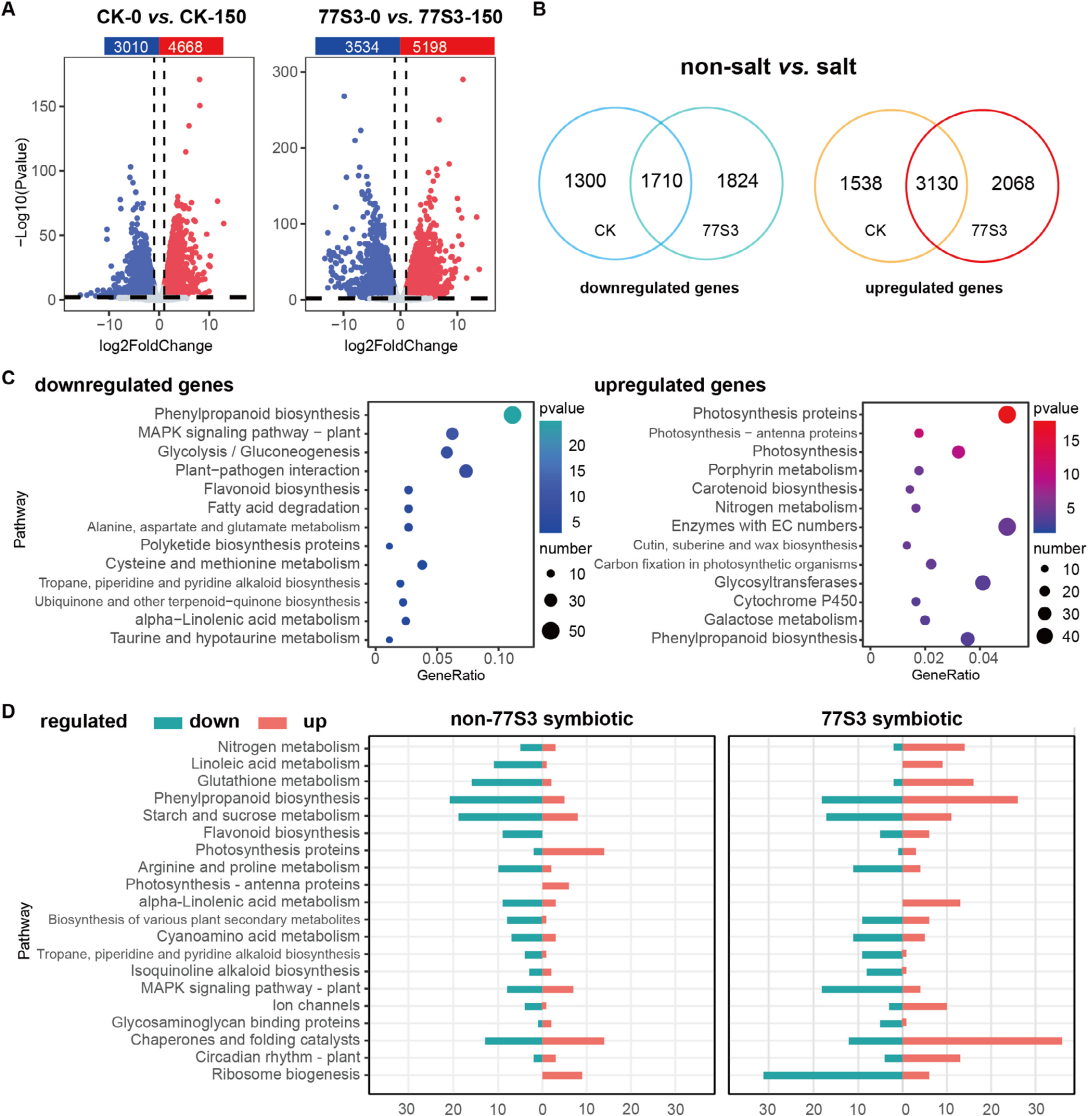
Transcriptional reprogramming in soybean roots induced by 77S3 and salt stress. (A) Volcano plots showing DEGs (|log_2_ (fold change)| ≥ 1, FDR < 0.05) in soybean roots under non‐salt and 150 mM NaCl salt stress, comparing 77S3‐symbiotic and non symbiotic samples. The *x*‐axis represents gene expression fold change, and the *y*‐axis represents mean gene expression values. Red dots denote significantly up‐regulated genes, and blue dots denote down‐regulated genes. Corresponding gene counts are shown as bar plots above the scatter plots. (B) Venn diagram of salt‐responsive DEGs shared and unique to 77S3‐symbiotic and control (non‐symbiotic) conditions. A total of 3130 up‐regulated and 1710 down‐regulated genes were common to both groups under salt stress. The 77S3‐symbiotic condition exhibited 2068 uniquely up‐regulated and 1824 uniquely down‐regulated genes, whereas the control condition showed 1538 uniquely up‐regulated and 1300 uniquely down‐regulated genes (*p* = 0.00e‐60). (C) Bubble plots of KEGG pathway enrichment analysis for down‐regulated (blue) and up‐regulated (red) genes in response to salt stress, independent of 77S3 symbiosis status. Bubble size represents gene number, and colour represents −log_10_ (*p*‐ value). (D) Bar plots showing up‐regulated (red) and down‐regulated (blue) metabolic processes in response to 77S3 symbiosis under non‐77S3 symbiotic (left) and 77S3‐symbiotic (right) conditions. The *y*‐axis lists KEGG functional categories, and the *x*‐axis indicates gene number. Complete gene lists and KEGG terms are provided in Table S7.

RNA‐Seq analysis revealed a pronounced reprogramming of the soybean root transcriptome under salt stress. 4668 genes were up‐regulated and 3010 down‐regulated in non‐inoculated roots (CK‐150 vs. CK‐0), while 5198 were up‐regulated and 3534 down‐regulated in 77S3‐inoculated roots (77S3‐150 vs. 77S3‐0) (Figure 4A, Tables S5 and S6). A core set of 3130 up‐regulated and 1710 down‐regulated genes responded to salt stress under both conditions, indicating a conserved transcriptional response (Figure 4B). Furthermore, 2068 up‐ and 1824 down‐regulated genes were unique to the 77S3‐symbiotic condition, compared to 1538 up‐ and 1300 down‐regulated under mock treatment (Figure 4B), demonstrating distinct regulatory patterns associated with inoculation (Figure 4B).

Functional enrichment analysis (KEGG and GO) of shared and condition‐specific DEGs revealed key pathways modulated by salt stress (Tables S7 and S8). Core salt‐responsive genes, independent of 77S3 inoculation, showed strong upregulation of photosynthesis‐related processes (e.g., photosystems, antenna proteins, carbon fixation), porphyrin and carotenoid metabolism, and nitrogen assimilation, and cutin/suberin/wax biosynthesis, supporting energy production and structural integrity (Figure 4C and Figure S4A). Osmoprotectant synthesis pathways, such as arginine/proline and glycosyltransferase‐mediated metabolism, were also induced. In contrast, down‐regulated genes were enriched in defence signalling (e.g., plant–pathogen interaction, MAPK signalling) and central metabolic processes including glycolysis/gluconeogenesis and amino acid metabolism (Figure 4C and Figure S4A). These results suggest that salt stress triggers a conserved reprogramming that reinforces photosynthetic capacity, structural integrity, and osmotic adjustment, while reducing energy‐consuming defence and metabolic pathways. This reallocation of resources supports a well‐organised strategy that helps plants adapt to stress.

Analysis of condition‐specific DEGs reveals distinctive molecular reprogramming under 77S3 symbiosis during salt stress. In inoculated roots, key upregulated pathways included nitrogen metabolism, phenylpropanoid biosynthesis, linoleic/α‐linolenic acid metabolism, glutathione metabolism, molecular chaperones, ion channels, and circadian regulation (Figure 4D and Figure S4C). This multi‐faceted response enhances nitrogen assimilation, reinforces membrane integrity through unsaturated lipids, bolsters antioxidative capacity via glutathione, maintains proteostasis, and regulates ion transport, which collectively mitigate oxidative and ionic stress. Conversely, down‐regulated genes were enriched in energy‐intensive processes, including ribosome biogenesis, alkaloid biosynthesis, cyanogenic amino acid metabolism, MAPK signalling, and secondary metabolite synthesis, indicating suppression of translational machinery and excessive defense responses (Figure 4D and Figure S4C). GO annotations support these findings: downregulation of ribosomal subunits and tRNA processing indicates reduced protein synthesis, while suppression of cell wall biogenesis and xylan metabolism suggests limited polysaccharide expansion. A shift in antioxidant strategy, from peroxidase‐based to glutathione‐mediated pathways, was observed, alongside dampened ethylene and histidine/receptor kinase signalling, reflecting deliberate attenuation of energy‐costly stress responses to reduce metabolic burden and avoid overreaction.

Collectively, 77S3 symbiosis promotes salt tolerance through improved nitrogen assimilation, ion transport, and optimised resource allocation. Non‐inoculated roots under salt stress showed minimal adaptive regulation and broad suppression of critical stress‐response pathways, underscoring the essential role of 77S3 in system‐level reprogramming.

### 2.5 77S3 Inoculation Alters Soybean Root Metabolism Under Salt Stress

To assess the metabolic impact of 77S3 inoculation, we conducted untargeted metabolomic analysis on soybean roots under salt stress and control conditions. Principal component analysis (PCA) showed tight clustering of quality control (QC) samples, confirming data reliability. The first two principal components (PC1 and PC2) explained 84.6% of the metabolic variance, with clear separation among the four experimental groups (Figure S5A). Significant metabolic alterations were induced by 77S3 inoculation regardless of salt stress. Orthogonal partial least squares‐discriminant analysis (OPLS‐DA) further distinguished metabolic profiles between inoculated and non‐inoculated plants under both conditions (Figure S5B). Using thresholds of VIP > 1.0 and |log_2_FC| ≥ 1, we identified 60 differentially expressed metabolites (DEMs) under control conditions (35 up‐ and 25 down‐regulated) and 92 under salt stress (28 up‐, 64 down‐regulated; Tables S9 and S10).

KEGG enrichment analysis revealed seven significantly enriched pathways specifically under salt stress (Table S11), including flavonoid biosynthesis, amino acid biosynthesis, and purine metabolism (Figure S5C). Notably, there were no significant enrichments observed in these pathways under normal conditions. These results demonstrate that 77S3 inoculation reprograms metabolic pathways related to flavonoids, amino acids, and purines in soybean roots under salt stress, highlighting its role in improving salt tolerance.

### 2.6 Integrated Transcriptomic and Metabolomic Analysis of Soybean Roots Under Salt Stress Induced by 77S3 Inoculation

**FIGURE 5:**
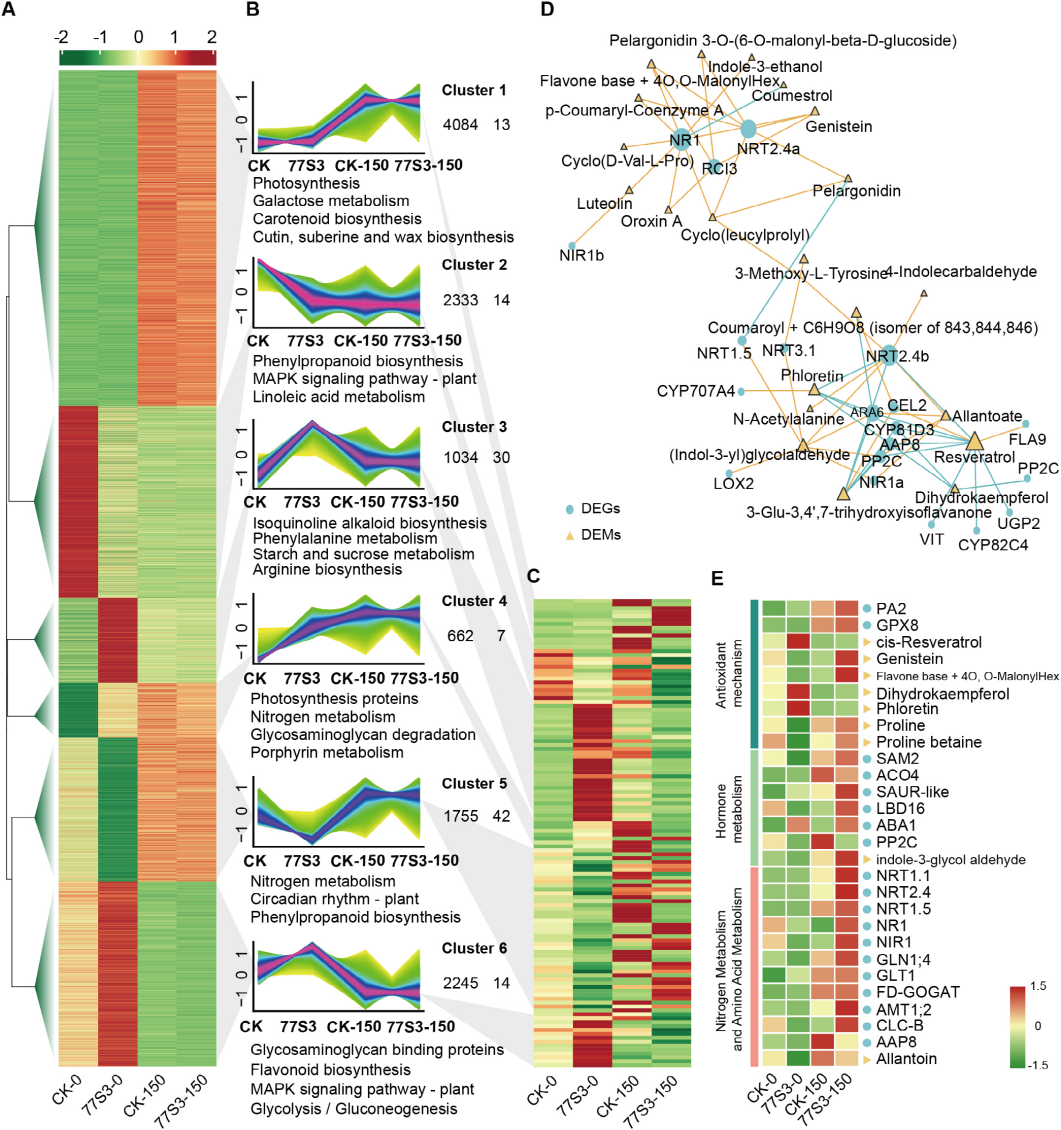
Integrated transcriptome–metabolome analysis of soybean root responses to 77S3 and salt stress. (A, C) Clustered expression profiles of up‐regulated and down‐regulated genes (A) and metabolites (C) in soybean roots inoculated with or without *Pseudomonas* sp. 77S3 under control (0 mM NaCl) and salt‐stress (150 mM NaCl) conditions, as revealed by integrated transcriptomic and metabolomic analyses. Heatmaps represent normalised gene expression levels (A) and relative metabolite abundances (C), respectively. (B) Line plots display co‐expression clusters and dynamic trends of transcripts and metabolites. Individual trajectories are shown in light lines, with the cluster centroid highlighted in red. Significantly enriched KEGG pathways (*q*‐value < 0.05) are indicated for each cluster. (D) Correlation network between DEGs (circles) and DEMs (triangles) with a correlation coefficient greater than 0.9 and a *p*‐value less than 0.01. Red and blue edges represent positive and negative correlations, respectively. (E) Heatmap visualisation of gene expression (circles) and metabolite abundance (triangles) across four conditions: CK‐0, 77S3‐0, CK‐150, and 77S3‐150. Values are presented as −log_2_(FPKM) for genes and log_2_(fold change) for metabolites.

To elucidate the mechanism of 77S3‐induced salt tolerance, we integrated root transcriptome and metabolome datasets and grouped DEGs and DEMs into eight hierarchical clusters, followed by KEGG enrichment analysis (Figure 5A–C). Cluster 1 represented a conserved salt‐responsive program (4084 DEGs and 13 DEMs) that was largely unaltered by 77S3 inoculation and enriched in pathways related to photosynthesis, carbohydrate metabolism, and cutin/membrane biosynthesis, consistent with typical plant adaptation to salinity (Figure 5B). In contrast, multiple clusters exhibited symbiosis‐specific expression patterns. Notably, Cluster 4 exhibited a symbiosis‐enhanced response, wherein genes and metabolites involved in photosynthetic antenna proteins, porphyrin/chlorophyll metabolism, and importantly, nitrogen uptake and assimilation pathways were upregulated by 77S3 and further amplified under combined 77S3 and salt conditions (Figure 5B). This was accompanied by elevated levels of redox‐related metabolites, indicating improved cellular redox homeostasis under such conditions. Other 77S3‐responsive clusters highlighted enhanced amino acid and carbohydrate metabolism, along with increased biosynthesis of antioxidant secondary metabolites, collectively supporting more effective mitigation of osmotic and oxidative stress (Figure 5B). Taken together, these multi‐omics results support a model wherein 77S3 augments photosynthetic energy capture and nitrogen assimilation while reinforcing antioxidant capacity and redox balance, thereby alleviating salt‐induced growth impairment. Based on these findings, we focused subsequent analyses on nitrogen assimilation and its associated redox regulation.

To further investigate the symbiosis‐specific effects of strain 77S3 under salt stress, we analysed correlations between DEGs and DEMs. A total of 76 DEGs were significantly correlated with 22 DEMs (absolute correlation coefficient > 0.8, *p* < 0.01; Table S12). To gain deeper insights into the regulatory mechanisms, a high‐confidence network (pearson correlation coefficient > 0.9) was constructed, comprising 19 genes and 21 metabolites with 41 positive and 21 negative correlations (Figure 5D). Key nitrogen metabolism encoding genes emerged as central nodes, including nitrate reductase 1 (*NR1*, *Glyma.13G084000*), nitrate transporters 2.4a (*NRT2.4a*, *Glyma.11G195200*), *NRT2.4b* (*Glyma.12G176900*), *NRT3.1* (*Glyma.05G042200*), *NRT1.5* (*Glyma.05G070600*), and nitrite reductases 1a (*NIR1a*, *Glyma.02G132100*) and *NIR1b* (*Glyma.07G212800*), each correlated with at least one metabolite. RT‐qPCR validation confirmed that salt stress significantly upregulates *NRT1.1* (*Glyma.11G031500*), *NRT2.4a*, and *NRT3.1* in inoculated roots (Figure S6B). These results demonstrate the key role of nitrogen metabolism in 77S3‐induced salt tolerance.

Furthermore, several critical genes were identified as central network components, including reactive oxygen species‐related *RCI3* (*Glyma.09G022800*), chloroplast thiazole biosynthetic enzyme *ARA6* (*Glyma.20G142000*), cytochrome P450 CYP81D3 (*Glyma.09G049300*), amino acid permease 8 (*AAP8*, *Glyma.06G088300*), protein phosphatase 2C (*PP2C*, *Glyma.06G050900*), and cellulase 2 (*CEL2*, *Glyma.18G030700*). These proteins are implicated in antioxidant production, thiamine biosynthesis, cytochrome P450 activity, amino acid transport, abscisic acid (ABA) signalling, and cellulose synthesis, and all these processes are activated by 77S3 inoculation to enhance stress resistance. Additionally, several metabolites served as core network nodes, each correlating significantly with three or more genes. Notably, allantoate, (indol‐3‐yl) glycolaldehyde, 3‐methoxy‐L‐tyrosine, genistein, and a flavone derivative (base+4O, O‐malonylHex) showed strong positive correlations, whereas phloretin, resveratrol, 3‐Glu‐3,4′,7‐trihydroxyisoflavanone, and a coumaroyl conjugate exhibited negative correlations. These metabolite associations implicate flavonoid, tryptophan, auxin, and uracil metabolism in 77S3‐mediated transcriptional reprogramming.

### 2.7 77S3 Enhances Salt Adaptation in Soybean Through *NRT1.5*‐Mediated Remodelling of Nitrogen Metabolism

**FIGURE 6:**
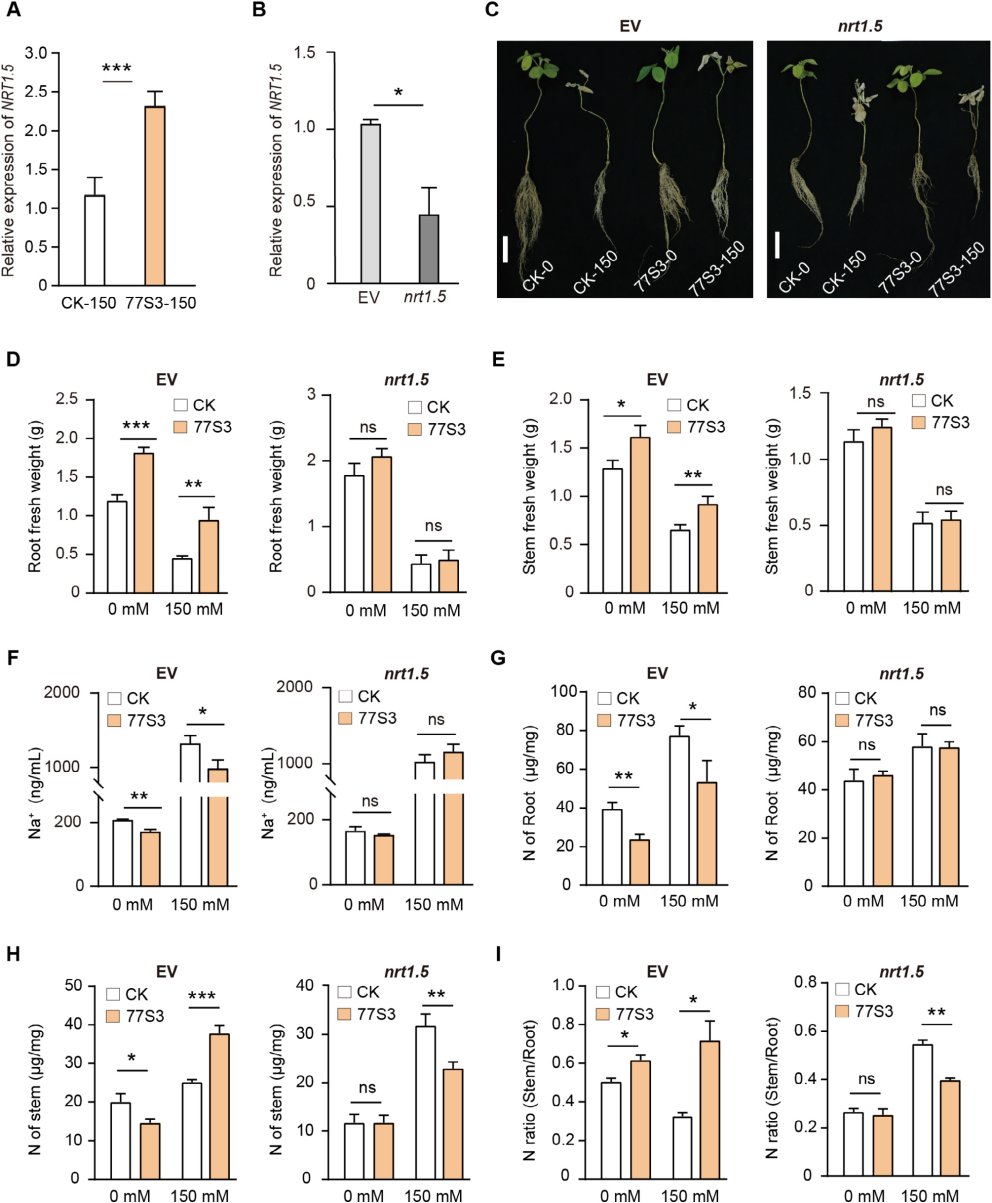
Functional characterisation of *GmNRT1.5* in *Pseudomonas* sp. 77S3‐mediated salt tolerance in soybean. (A and B) *GmNRT1.5* expression analysis by RT‐qPCR. Relative transcript levels in roots of non‐inoculated (CK) versus 77S3‐inoculated plants under salt stress (A). Expression in hairy roots transformed with empty vector (EV) or the *nrt1.5* RNAi construct (B). All data were normalised to the endogenous reference gene *GmTUB2*. (C) Phenotypic responses of soybean plants across different genetic and inoculation treatments under 0 mM and 150 mM NaCl. Scale bar = 5 cm. (D–I) Physiological parameters of EV‐ and *nrt1.5*‐expressing hairy roots with or without 77S3 inoculation under 0 or 150 mM NaCl: Root fresh weight (D), stem fresh weight (E), root Na^+^ content (F), root nitrogen concentration (G), stem nitrogen concentration (H), and stem‐to‐root nitrogen allocation ratio (I). (A–B, D–I) Data are shown as mean ± SD (*n* = 3). Significance was determined by two‐tailed Student's *t*‐test: *p < 0.05, **p < 0.01, ***p < 0.001; ns, not significant.

Integrated omics analyses revealed that 77S3 inoculation enhances nitrogen uptake and assimilation under salt stress (Figure S3B,D and Figure 5B,D). Key nitrate transporters *NRT1.1* and *NRT2.4* were upregulated approximately 7‐fold and 4‐fold, respectively, alongside elevated expression of *NRT1.5*, indicating broader activation of nitrogen assimilation pathways (Figures 5E, 6A). Strain 77S3 also alleviated the negative impact of high salt on nitrogen assimilation by upregulating *NR* genes, which are essential for nitrate reduction, by approximately 3.6‐fold (Figure 5E). This upregulation was accompanied by increased expression of *NIR* and glutamate synthase (*GLT1*, *Glyma.04G236900* and *FD‐GOGAT*, *Glyma.03G128300*), enzymes critical for nitrogen metabolism and ammonia assimilation (Figure 5E). Additionally, upregulation of ammonium transporter 1;2 gene (*AMT1;2*, *Glyma.10G167800*) and chloride ion channel‐b (*CLC‐b*, *Glyma.19G089800*) further reflects a coordinated adaptation to salinity (Figure 5E). Given the significant impact of strain 77S3 on the transcriptional regulation of nitrogen metabolism under stress, we sought to determine whether these molecular changes translate into physiological improvements in nitrogen allocation. Nitrogen concentration measurements in symbiotic roots provided clear physiological validation: under salt stress, inoculation with 77S3 promoted nitrogen translocation from roots to shoots, resulting in a significant decrease in root N content and a concurrent increase in shoot N content (Figure S6D). Although the redistribution pattern was observable under control conditions (Figure S6C), this effect was significantly less pronounced, indicating a role of 77S3 in amplifying host nitrogen partitioning, particularly under saline stress. Collectively, these findings demonstrate that strain 77S3 plays a crucial role in optimising nitrogen allocation under salt stress.

To functionally validate the role of nitrogen transport in 77S3‐mediated salt tolerance, we focused on the key transporter *NRT1.5* (Lin et al. 2008; Meng et al. 2016). The expression of *NRT1.5* was induced by 77S3 inoculation under salt stress (Figures 5E and 6A). Using RNAi, we generated soybean roots with knocked‐down *NRT1.5* expression (hereafter referred to as *nrt1.5* lines) (Figure 6B,C). Under normal conditions, *nrt1.5* mutants exhibited a slightly weaker growth phenotype with mild leaf yellowing, likely resulting from impaired nitrogen transport (Figure 6C). Under 150 mM salt stress, empty vector (EV) controls inoculated with 77S3 showed improved growth, whereas *nrt1.5* mutants failed to develop salt tolerance even upon 77S3 inoculation (Figure 6C). Salt stress significantly reduced shoot and root fresh weights in EV plants, increased Na^+^, and decreased K^+^ content (Figure 6D–F; Figure S6E). Inoculation with 77S3 mitigated these effects in EV plants, with an increase in biomass and a reduction in Na^+^. By contrast, 77S3 inoculation did not alter fresh weights or ion contents in *nrt1.5* mutants under salt stress (Figure 6D–F). Furthermore, under both control and salt‐stress conditions, inoculation with 77S3 significantly reduced root N content in empty vector (EV) plants while markedly increasing N accumulation in the shoots, an effect that was particularly pronounced under salinity (Figure 6G,H). By contrast, this shift in N allocation was abolished in *nrt1.5* mutants (Figure 6G,H). Together, these results underscore the essential role of *NRT1.5* in mediating symbiosis‐driven translocation and redistribution of nitrogen to the shoot, which ultimately led to a significantly elevated shoot‐to‐root N ratio (Figure 6I).

Collectively, these results demonstrate that *NRT1.5* is essential for 77S3‐mediated salt tolerance, likely through reprogramming nitrogen allocation and ion homeostasis. We thus propose that 77S3‐induced remodelling of nitrogen metabolism enhances salt adaptation by coordinating nitrogen balance, antioxidant capacity, and stress‐related signalling.

## 3 Discussion

Plant endophytes are symbiotic microbes that significantly enhance plant growth and stress tolerance against salinity, drought, and pathogens (Liu et al. 2023). Among these beneficial microorganisms, plant growth‐promoting rhizobacteria (PGPR) are crucial for sustainable agriculture, particularly in alleviating salt‐alkali stress. However, the specific roles of key endophytes and potential ‘plant‐microbe functional combinations’ remain inadequately explored (Trivedi et al. 2020; Zhang et al. 2024).

In this study, we isolated strain 77S3, a root endophytic bacterium from salt‐tolerant wild soybeans, which exhibits multiple plant‐beneficial traits including nitrogen fixation, IAA production, siderophore synthesis, and phosphorus‐potassium solubilisation (Han et al. 2020; Perez‐Alonso et al. 2022; Ren et al. 2009; Ren and Dai 2013). Strain 77S3 improved soybean germination, promoted growth, and enhanced salt stress resistance, serving as an ideal model for investigating endophyte‐mediated stress tolerance mechanisms. Our multi‐omics analyses revealed sophisticated molecular interactions between 77S3 and soybeans, demonstrating that this endophyte enhances growth and salt tolerance by regulating gene expression and metabolic reprogramming, with a pronounced effect on nitrogen metabolism.

### 3.1 77S3 Symbiosis Promotes Soybean Growth and Salt Stress Tolerance

To investigate the symbiotic mechanisms of the beneficial endophyte 77S3, we used cultivated soybean roots as a model system. Colonisation assays demonstrated that 77S3 stably colonises the meristematic and elongation zones of root apices (Figure 3C), confirming active plant‐microbe interactions. Future studies may explore its entry mechanism in greater detail, such as via root fissures or intercellular penetration. Under non‐stress conditions, 77S3 symbiosis significantly promoted soybean growth, increasing plant height, chlorophyll content, and root development. Under salt stress, these benefits were more pronounced, with improved root length, higher chlorophyll retention, reduced leaf abscission, and increased biomass, collectively enhancing plant vigour and stress resilience (Figure 3B). These results suggest that 77S3 establishes a synergistic plant‐microbe partnership that improves plant performance through multifaceted mechanisms.

### 3.2 77S3 Enhances Salt Adaptation Through Transcriptional Reprogramming

Salt stress impairs plant growth by causing osmotic imbalance, ion toxicity, and nutrient deficiency, ultimately leading to cellular damage. Although PGPR are known to alleviate salt stress and promote plant growth, the molecular mechanisms behind these beneficial effects remain poorly understood and often strain‐ and host‐specific. Under salt stress, core DEGs in soybeans revealed premature senescence due to sodium toxicity, reduced photosynthetic efficiency, and disrupted metabolism (Figure 4 and Figure S3). However, 77S3 inoculation effectively mitigated these inhibitory effects. Condition‐specific DEG analysis revealed that 77S3 symbiosis coordinates nitrogen assimilation and transport, thereby maintaining metabolic homeostasis under salinity (Figure 4C,D and Figure S3C,D).

This facilitation of nutrient and ion uptake helps stabilise photosynthesis and metabolism, improving salt adaptability. Integrated transcriptomic and metabolomic analyses supported these findings, with co‐expression cluster 4 highlighting the 77S3‐specific enhancement of energy–nitrogen supply and redox balance in salt‐stressed roots (Figure 5B). Furthermore, co‐expression networks emphasised the central role of nitrogen metabolism genes in coordinating metabolic adaptation under salt stress (Figure 5D).

### 3.3 Nitrogen Metabolism Reprogramming by 77S3 Symbiotic Enhances Soybean Salt Tolerance

Nitrogen is a fundamental nutrient for plant growth, integral to nucleic acids, proteins, chlorophyll, and stress‐responsive metabolites (Shi et al. 2022). High salinity disrupts nutrient uptake and impairs nitrogen metabolism, while nitrogen supplementation is known to mitigate salt stress (Alfatih et al. 2023). In legumes like soybean, nitrogen acquisition involves interactions with arbuscular mycorrhizal fungi, rhizobia, and other microbes capable of fixing atmospheric N_2_. Ammonia‐oxidising bacteria further facilitate the conversion of ammonium (${\mathrm{NH}}_{4}^{+}$) to nitrate (${\mathrm{NO}}_{3}^{-}$), which is absorbed via root *NRT* and ammonium (AMT) transporters (Richardson et al. 2009). Multi‐omics analyses revealed that 77S3 symbiosis reprograms nitrogen metabolism in soybean roots, enhancing salt adaptation. Specifically, under non‐salt stress conditions, 77S3 down‐regulated genes encoding external nitrogen transport proteins (*NRT1.1*, *NRT2.1*), but upregulated *NRT1.2* and *NRT1.5*, facilitating nitrate allocation to the cortex and xylem (Figure 5E). Furthermore, strain 77S3 also demonstrated nitrogen‐fixing capacity, suggesting it may contribute directly to nitrate supply and enhance rhizosphere nitrogen transport mobility.

Consistent with these mechanistic insights, quantitative analysis of nitrogen levels revealed that, under salt stress, 77S3 symbiosis significantly reduced nitrogen content in the roots while increasing it in the shoots (Figure 5G–I and Figure S6D). This result demonstrates that the symbiosis facilitates a coordinated reprogramming of nitrogen metabolism by promoting nitrogen allocation to aerial tissues and its systemic redistribution under saline conditions. Functional validation using *nrt1.5* confirmed the essential role of this transporter in the symbiosis: mutants exhibited growth impairment and leaf chlorosis under normal conditions and failed to show 77S3‐induced growth promotion or salt tolerance (Figure 6B–E). Moreover, in *nrt1.5* mutants under salt stress, 77S3 inoculation failed to alter the Na^+^/K^+^ ratio, indicating a loss of the symbiosis‐induced ion homeostasis regulation (Figure 6F and Figure S6E). Consistent with this phenotype, both the symbiosis‐dependent nitrogen allocation pattern and the associated reduction in root nitrogen content were also abolished in the mutants (Figure 6I).

Together, these results establish that 77S3 enhances salt tolerance primarily via *NRT1.5*‐dependent nitrogen metabolic reprogramming.

### 3.4 77S3 Modulates Oxidative Stress and Hormonal Signalling Pathways

Symbiosis‐induced reprogramming of nitrogen metabolism enhances salt tolerance in soybean by coordinating carbon–nitrogen allocation, antioxidant capacity, and MAPK signalling, thereby supporting overall plant health (Al‐Turki et al. 2023; Hasanuzzaman et al. 2022). Salt stress disrupts ion homeostasis and promotes ROS accumulation, damaging cellular structures (Matsuo et al. 2015). Antioxidant systems are essential for ROS scavenging and stress adaptation. PGPR are known to enhance ROS detoxification under abiotic stress (Chen et al. 2020; Handa et al. 2019). Inoculation with strain 77S3 under salinity significantly up‐regulated genes encoding key antioxidant enzymes, including glutathione peroxidase 8 (*GPX8*, *Glyma.01G219502*) and peroxidase 2 (*PA2*, *Glyma.01G171100*), mitigating ROS accumulation and oxidative damage (Figure 5E). Additionally, it enhanced the accumulation of nonenzymatic antioxidants such as resveratrol, flavonoids, proline, and betaine, which function as osmoprotectants and radical scavengers (Figure 5E). This synergistic enhancement of enzymatic and nonenzymatic defences is critical for reducing oxidative cellular damage under stress (Kusale et al. 2021) (Figure 5E).

Plant hormones play integral roles in mediating environmental stress responses. PGPR enhance plant growth by metabolising tryptophan and exudates to produce phytohormones including auxins, gibberellins, and cytokinins. Notably, auxin, especially IAA, plays an essential role in regulating root architecture (Kim et al. 2014). In this study, 77S3 influences auxin biosynthesis by elevating indole‐3‐acetaldehyde (Figures 1E and 5E), potentially facilitating transport via NRT1.1 and promoting root development. Under salt stress, the expression of S‐adenosylmethionine synthase 2 (SAM‐2, Glyma.10G144300), which participates in the synthesis of the ethylene precursor 1‐aminocyclopropane‐1‐carboxylic acid (ACC) (Naing et al. 2021), was upregulated. This induction was further enhanced by inoculation with strain 77S3 (Figure 5E). In contrast, the ethylene biosynthesis gene ACO4 (Glyma.07G264200), which converts ACC to ethylene, was down‐regulated during symbiosis (Figure 5E). This led to ACC accumulation without proportional ethylene production. We hypothesize that 77S3 may secrete ACC deaminase (ACCD), degrading ACC and attenuating ethylene signalling, thereby alleviating its growth‐inhibitory effects, consistent with earlier reports on endophyte‐mediated stress mitigation via ACCD (Elías et al. 2018). Additionally, under high‐salt stress, 77S3 also upregulated key ABA biosynthesis and signalling genes, including (*ABA1*, *Glyma.09G000600*) and protein phosphatase 2C (*PP2C*, *Glyma.17G230600*, *Glyma.14G093002*), indicating enhanced ABA‐mediated stress adaptation (Figure 5E) (Li et al. 2019). Auxin‐responsive genes such as SAUR‐like (*Glyma.03G029600*) and LATERAL ORGAN BOUNDARIES‐DOMAIN 16 (*LBD16*, *Glyma.03G161400*) were also upregulated, supporting lateral root formation and architectural adaptation to salinity (Figure 5E).

Beyond their role in nitrate transport, NRT/NPF family proteins are known to facilitate the movement of a broad spectrum of substrates, including hormones such as abscisic acid (Zhang et al. 2022), auxin (Watanabe et al. 2020), and gibberellins (Binenbaum et al. 2023), as well as specialised metabolites like α‐tomatine (Kazachkova et al. 2021) and glycerate (Lin and Tsay 2023). This functional versatility suggests that nitrate transporters may play integral roles in coordinating complex physiological responses. In line with this, our study reveals that the transcriptional reprogramming induced by the endophyte *Pseudomonas* sp. 77S3 is closely linked to nitrogen signalling components, particularly *NRT1.5*, underscoring its essential role in enhancing root plasticity and salt tolerance in soybean. We demonstrate that *NRT1.5*‐mediated mechanisms extend well beyond conventional nitrogen allocation and assimilation. Under salinity stress, *NRT1.5* likely contributes to ion homeostasis, a function corroborated by emerging evidence that certain NRT‐type transporters are involved in ion transport (Wu et al. 2025). Moreover, the extensive rewiring of phytohormone pathways in 77S3‐inoculated plants suggests that *NRT1.5* may interface with hormonal signalling to fine‐tune stress adaptation. Significantly, this hypothesis is reinforced by recent work demonstrating that the rice ortholog NRT1.1B acts as an ABA receptor, enabling cross‐talk between nitrate sensing and ABA‐mediated stress responses (Ma et al. 2025). Thus, *NRT1.5* appears to function as a multimodal integrator of nutrient, ion, and hormone signals, highlighting its central role in symbiotic stress acclimation and positioning it as a key player in microbial‐mediated improvement of crop resilience.

In summary, the wild soybean‐derived endophyte *Pseudomonas* sp. 77S3 enhances soybean salt tolerance via multi‐level molecular reprogramming involving nitrogen fixation, ACC deaminase activity, auxin biosynthesis, and nutrient solubilisation. This symbiosis depends on the nitrate transporter *NRT1.5*, which serves as a regulatory hub for nitrogen‐dependent rebalancing of nitrogen allocation, redox homeostasis, and stress signalling. These findings uncover a synergistic plant–microbe interaction that improves root architecture and metabolic resilience under salinity, providing novel insights into the mechanisms of how endophytes reprogram host physiology to enhance abiotic stress tolerance.

## 4 Conclusion

**FIGURE 7:**
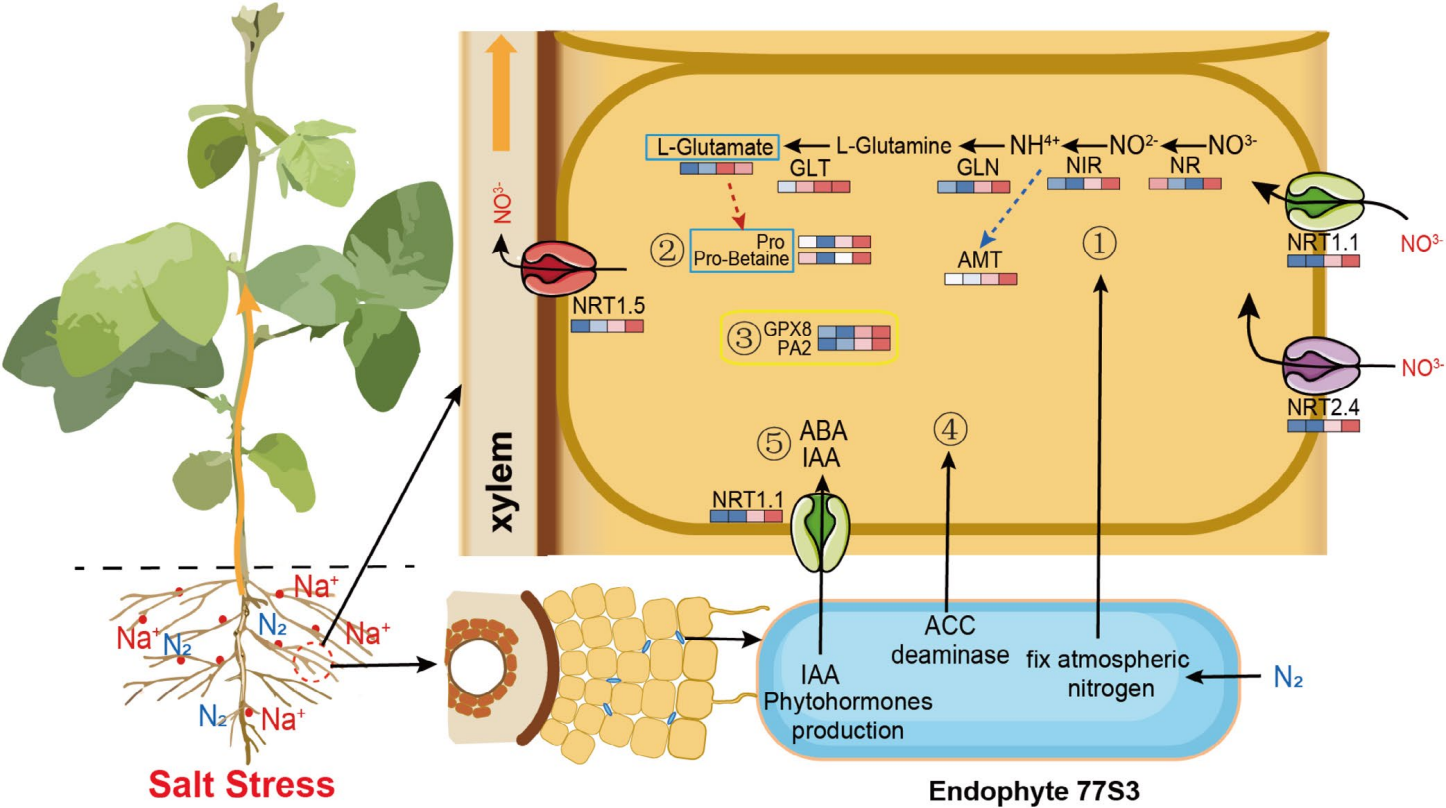
Proposed model for endophyte *Pseudomonas* sp. 77S3‐mediated salt tolerance in soybean. This schematic illustrates the molecular and physiological mechanisms through which *Pseudomonas* sp. 77S3 enhances salt adaptation in soybean. Under salinity stress, 77S3 inoculation systemically reprograms host gene expression and metabolic pathways, leading to improved growth and stress resilience. Key mechanisms include enhanced nitrogen fixation and assimilation through upregulation of nitrate transporters (*NRT1.1*, *NRT1.5*, *NRT2.4*) and metabolic enzymes (*NR*, *NiR*, *GLN*, *GLT*, *AMT*); accumulation of osmoprotectants such as proline and proline‐betaine; reinforcement of antioxidant defence via increased activity of enzymes including *GPX8* and *PA2*; modulation of phytohormone signalling through ACC deaminase activity and changes in IAA and ABA pathways; and improved ion homeostasis mediated by *NRT1.5*‐dependent nitrate and sodium transport. Blue boxes denote metabolite changes, and heatmaps represent gene expression or metabolite abundance across conditions: CK‐0, 77S3‐0, CK‐150, and 77S3‐150, with values presented as log_2_‐transformed FPKM or relative abundance. These coordinated responses collectively enhance salt tolerance, supporting the use of 77S3 as a sustainable bioinoculant in saline agriculture.

Our study reveals that the wild soybean endophyte *Pseudomonas* sp. 77S3 enhances salt tolerance in soybean through extensive transcriptional and metabolic reprogramming. *NRT1.5*‐mediated nitrogen remodelling is essential for this symbiosis, enabling efficient nitrogen reallocation and restoring ion and redox homeostasis under salinity (Figure 7). Furthermore, 77S3 fine‐tunes phytohormone signalling through auxin biosynthesis, ACC deaminase‐mediated ethylene modulation, and ABA‐responsive pathways, collectively optimising root architecture and stress resilience (Figure 7). These findings provide novel insights into how beneficial microbes reprogram host physiology to combat abiotic stress and underscore the potential of 77S3 as a promising bioinoculant for improving crop productivity in saline‐alkaline agriculture.

## 5 Materials and Methods

### 5.1 Plant Materials and Growth Conditions

Wild soybean ( *Glycine soja* ) plants were collected from saline‐alkaline soils in Dongying, Shandong Province, China, for the isolation of endophytic bacteria. The cultivated soybean ( *Glycine max* ) cultivar Williams 82 was used as the host plant throughout this study. Seeds were surface‐sterilised and germinated under sterile conditions. Plants were grown in a greenhouse maintained at 25°C under a 16‐h‐light/8‐h‐dark photoperiod.

### 5.2 Endophyte Screening From Wild Soybean

Endophytes were isolated from the roots, stems, and leaves of healthy salt‐tolerant wild soybean plants. Fresh tissue samples (2 g) were rinsed under running tap water for 30 min, surface‐sterilised by immersion in sterile water (30 s, three repetitions), 75% ethanol (3 min), and 1% NaClO (3 min), followed by three rinses with sterile water. The samples were then stored at 4°C in darkness. Sterilisation efficacy was confirmed by plating the final rinse water on NA medium. Surface‐sterilised tissues were macerated with a sterile razor blade, homogenised using a mortar and pestle with quartz sand and 5 mL of sterile saline, and serially diluted with sterile water to obtain endophyte suspensions. Aliquots of diluted suspensions were spread on NA plates and incubated at 30°C for 24 h in darkness. Morphologically distinct bacterial colonies were selected, purified via streak plating, activated at 30°C, and preserved in glycerol at −80°C for long‐term preservation.

### 5.3 Assessment of Endophyte Salt Tolerance and Strain Identification

Endophytes were cultured on beef extract peptone solid medium supplemented with NaCl at concentrations of 200, 300, or 500 mM, and pH values of 7.0 or 9.0. Salt tolerance was assessed based on growth after 48 h incubation at 30°C.

Genomic DNA was extracted from the dominant strains for subsequent molecular characterisation. The 16S rRNA gene was amplified using universal primers 27F and 1492R (Table S1), and the resulting sequences were aligned against the NCBI database for preliminary phylogenetic assignment. Phylogenetic analysis was performed in MEGA 7.0 using the neighbour‐joining method. For precise taxonomic identification, whole‐genome draft sequencing was subsequently performed. The draft genome was assembled and taxonomically classified using the GTDB‐Tk classify_wf workflow. The genome draft sequence has been deposited in the GWH database (accession: GWHHAGN00000000.1). Cellular morphology was characterised through Gram staining and light microscopy.

### 5.4 Assessment of Growth‐Promoting Properties of Endophytes

The PGP properties of *Pseudomonas* sp. 77S3 were assessed through nitrogen fixation, phosphorus and potassium solubilisation, IAA, and siderophore production. Nitrogen fixation was evaluated on Burk's medium. Nitrogenase activity was assessed using the acetylene reduction assay (ARA) (Bellenger et al. 2014). The nitrogen fixation gene *nif*H was detected with universal primers (Table S1). Strain 77S3 was grown in nitrogen‐free liquid medium, and during the logarithmic growth phase, cultures were transferred to sealed 100 mL vessels. The headspace atmosphere was adjusted by replacing 10% of the air with high‐purity acetylene. The cultures were then incubated at 28°C with shaking at 240 rpm. Ethylene concentration was measured by gas chromatography after 12 and 24 h. Uninoculated medium supplemented with acetylene served as the negative control. Nitrogenase activity was expressed as the ethylene production rate (nmol/mL/h). Phosphorus solubilisation was tested on NBRIP medium, and solubilisation efficiency was calculated as the dissolution ratio (D/d), where D is the diameter of the solubilisation zone and d is the colony diameter. Potassium dissolution was evaluated on potassium bacteria agar medium (Hu et al. 2006). Siderophore secretion was detected using chrome azurol‐S (CAS) blue agar (Louden et al. 2011), and IAA production was quantified colorimetrically with the Salkowski assay (Glickmann and Dessaux 1995).

### 5.5 Bacterial Inoculum Preparation

Fresh inoculum was prepared as follows: *Pseudomonas* sp. 77S3 was cultured in 100 mL of NA liquid medium at 30°C with shaking at 180 rpm for 48 h. Bacterial cells were then harvested by centrifugation, washed three times with sterile water, and finally resuspended in sterile water to an optical density at 600 nm (OD_600_) of 1.0.

For the preparation of lyophilized inoculum, *Pseudomonas* sp. 77S3 was cultivated at large scale using an optimised medium composed of (per litre): yeast extract (18 g), yeast peptone (17 g), (NH_4_)_2_SO_4_ (3 g), KH_2_PO_4_ (0.7 g), K_2_HPO_4_ (0.7 g), MgSO_4_ (0.8 g), and defoamer (2 mL). During the mid‐fermentation phase, additional glucose (200 g), yeast extract (30 g), and yeast peptone (30 g) were supplemented. Following 48 h of fermentation at 37°C and 220 rpm, the bacterial density reached approximately 2.0 × 10^9^ CFU (colony‐forming units)/mL. Cells were collected by centrifugation, three‐times washed with sterile water and subjected to lyophilization. The resulting lyophilized powder had a viable bacterial count of approximately 2.5 × 10^10^ CFU/g, was stored at 4°C. Prior to application, the lyophilized bacterial powder was resuscitated in ultrapure water, washed three times with sterile water, and adjusted to an OD_600_ of 1.0, with viability confirmed throughout the process.

### 5.6 Germination and Salt Stress Assays

Surface‐sterilised soybean seeds were subjected to germination and seedling stress assays under controlled conditions. For germination analysis, seeds were soaked for 4 h in bacterial suspensions (OD_600_ = 1.0) or sterile water as control, placed on filter paper saturated with either 150 mM NaCl or pure water, and incubated at 25°C for 5 days before recording germination rates. For vermiculite‐based seedling assays, seeds were similarly treated with bacterial suspension or sterile water for 4 h, sown in sterilised, pre‐moistened vermiculite, and grown for 7 days. Seedlings were then assigned to four treatment groups: CK‐0 (non‐inoculated, sterile water), 77S3‐0 (inoculated with 77S3, sterile water), CK‐150 (non‐inoculated, 150 mM NaCl), and 77S3‐150 (inoculated with 77S3, 150 mM NaCl), with each group containing at least 20 plants and the experiment repeated in three independent biological replicates. For soil‐based evaluation of lyophilized inoculum, surface‐sterilised seeds were sown in sterilised potting soil. After 7 days, plants were assigned to the four standard treatment groups. Inoculated plants received reconstituted lyophilized bacterial suspension (OD_600_ = 1.0), followed two days later by salt treatment with 150 mM NaCl, while control plants were maintained with sterile water throughout the experiment. For functional validation using *nrt1.5* knockdown transgenic composite plants, individuals with GFP‐positive hairy roots (verified by RT‐qPCR) were transferred to vermiculite and acclimated for 3 days before treatment application. Plants were inoculated with bacterial suspension (OD_600_ = 1.0), followed two days later by exposure to 150 mM NaCl solution or sterile water for controls. All plants were harvested two weeks after salt initiation for phenotypic assessment and ion content analysis.

### 5.7 Generation of *nrt1.5* Mutant

To generate an RNAi‐mediated *nrt1.5* mutant, a 261‐bp fragment corresponding to the 5′ region of *GmNRT1.5* (positions +1 to +261 relative to the ATG start codon) was cloned into the RNAi vector pG2RNAi2 (He et al. 2021). Hairy root transformation was carried out as described previously (He et al. 2021). Briefly, surface‐sterilised soybean seeds were germinated on kraft paper. After germination, the hypocotyl was excised to remove the root, and the remaining shoot was retained for transformation. *Agrobacterium rhizogenes* strain K599 harbouring the constructs was applied to the wound site to induce hairy root development. Successful transformation was confirmed by GFP fluorescence, followed by RT‐qPCR analysis.

### 5.8 Determination of Physiological Parameters

Plant chlorophyll content (SPAD value) was determined using a SPAD‐502 chlorophyll meter (Konica Minolta, Japan), which operates based on Beer's law by quantifying chlorophyll absorption of light (Uddling et al. 2007). For mineral nutrient analysis, plant samples were dried at 65°C for 48 h, ground to a fine powder (200–300 mesh), and digested in concentrated nitric acid at high temperature (at 180°C for 1.5 h using an ETHOS UP microwave digestion system). Digested samples were diluted appropriately, and Na, K, and N contents were quantified using inductively coupled plasma triple quadrupole mass spectrometry (ICP‐MS/MS) and CHNS Analyzer, respectively.

### 5.9 Analysis of 77S3 Colonisation in Soybean Roots

The fluorescent reporter strain 77S3‐RFP was constructed by introducing plasmid pDSK‐RFPuv into strain 77S3 via electroporation. Soybean roots non‐inoculated or inoculated with 77S3‐RFP were sampled at 3, 10, and 20 days post‐inoculation. Root segments (0.5 cm) from different zones (crown, meristematic, elongation, and maturation) were fixed in 2.5% glutaraldehyde, mounted on slides, and observed under a confocal laser scanning microscope (Leica TCS SP3) with excitation at 558 nm and emission detection at 580–620 nm.

### 5.10 RNA Sequencing and Quantitative PCR

Total RNA was extracted from roots of the four treatment groups (77S3‐inoculated and mock‐treated plants, with or without salt stress) at 4 weeks post‐inoculation. RNA quality was assessed using a Nanodrop 8000 spectrophotometer and Bioanalyzer 4200. Libraries were constructed using the Hieff NGS Ultima Dual‐mode mRNA Library Prep Kit (Yeasen Biotechnology). Sequencing was performed on the MGI DNBSEQ T7 platform to generate 150 bp paired‐end reads. Raw data are deposited in the Genome Sequence Archive (Chen et al. 2021) at the National Genomics Data Center under accession number CRA016222 at https://ngdc.cncb.ac.cn/gsa. For real‐time quantitative PCR (qPCR), cDNA was synthesised using the SPARKscript II RT Plus Kit. Gene expression was analysed using SYBR Green on an ABI QuantStudio system (Applied Biosystems), and normalised to the constitutively‐expressed soybean *TUBULIN2* (*GmTUB2*) gene and calculated via the 2^−∆∆Ct^ method (Livak and Schmittgen 2001). Three biological replicates were included for each sample. Primers are listed in Table S1.

### 5.11 Analysis of Sequencing Data

Raw sequencing reads were quality‐trimmed and adapter‐filtered using Fastp (v0.12.4) (Chen et al. 2018). Kallisto (v0.46.0) (Bray et al. 2016) was used to align reads to the *Glycine max* reference genome (Wm82.a4.v1) (https://phytozome.jgi.doe.gov/) and quantify transcript abundance in TPM. Differential gene expression analysis was performed with edgeR (v3.22.5), with differentially expressed genes (DEGs) defined as |log_2_FC| ≥ 1 and FDR < 0.05. Functional enrichment of DEGs was analyzed via GO and KEGG using clusterProfiler (Yu et al. 2012).

### 5.12 Metabolomic Analysis

For metabolomic profiling, root tissue from 77S3‐inoculated and control plants under 0 or 150 mM NaCl were flash‐frozen in liquid nitrogen and stored at −80°C. Metabolites were extracted from 100 mg tissue using 80% methanol at 4°C overnight, filtered through 0.22 μm membranes, and centrifuged at 12 000 rcf (relative centrifugal force) for 10 min at 4°C. Untargeted LC–MS/MS analysis was performed. Metabolomic data are deposited in OMIX at the China National Center for Bioinformation under accession number OMIX006345 at https://ngdc.cncb.ac.cn/omix.

### 5.13 Data Preprocessing and Annotation

LC–MS data were processed using Compound Discoverer, with metabolites annotated against Human Metabolome Database (HMDB) and public databases. Principal component analysis (PCA) and partial least squares‐discriminant analysis (PLS‐DA) were employed to assess metabolic profile differences and data reliability (Yi et al. 2021). To screen for differential metabolites, an important variable in prediction (VIP) was applied. Differentially accumulated metabolites (DAM) were selected based on |log_2_FC| ≥ 1 or FDR ≤ 0.05 (Chen et al. 2013). Kyoto Encyclopedia of Genes and Genomes (KEGG) enrichment analysis was performed with a significance threshold of *p*.adjust ≤ 0.05.

### 5.14 Hierarchical Clustering and Co‐Expression Network Construction

Time‐series expression patterns of differentially expressed genes (DEGs) and metabolites (DEMs) were profiled using STEM (Ernst and Bar‐Joseph 2006) (www.omicshare.com/tools). An integrated co‐expression network was constructed via the Metware Cloud platform (https://cloud.metware.cn) (Bai et al. 2022), retaining strong (|*r*| > 0.8) and significant (*p* < 0.05) Pearson correlations. Nodes represent genes (circles) and metabolites (triangles), with edges coloured red (positive) or blue (negative) to indicate correlation direction.

## Author Contributions

Xiao Luo conceived the study. Xiao Luo, Wanying Zhang, Tianqi Wang and Chengyang Song designed the experiments. Wanying Zhang, Tianqi Wang, and Chengyang Song performed most of the experiments. Chengyang Song conducted bioinformatic analysis. Xiulin Liu, Yisheng Fang, Zhu Yan, Yaxi Zhu, Na Zheng, Xiaofei Ma, Guochen Qin, Dan Zhu, Junchuan Xiao performed part of the experiments; Xiao Luo, Wanying Zhang, Tianqi Wang, Chengyang Song, and Xing Wang Deng interpreted results. Xiao Luo wrote the paper with the help from Chengyang Song, Wanying Zhang, and Tianqi Wang.

## Funding

The work was supported in part by the National Key R&D Program of China (2024YFF1000303 to Xiao Luo), National Natural Science Foundation of China (32570416 to Xiao Luo), Taishan Scholars Program (tsqn202211301 to Xiao Luo), National High‐Level Talents Special Support Plan (to Xiao Luo), Natural Science Foundation of Shandong Province (ZR2021YQ16 to Xiao Luo, ZR2023QC085 to Yisheng Fang, ZR2022QC262 to Xiulin Liu, project SYS202206), Yuandou Scholars Program (to Xiao Luo), and the Excellent Research Group Project of the National Natural Science Foundation of China (Grant No. 32488102 to Xing Wang Deng).

## Conflicts of Interest

The authors declare no conflicts of interest.

## Supporting information


**Figure S1:** Identification of endophytic isolates and evaluation of their salt tolerance and plant growthpromoting effects. (A) Taxonomic distribution of the dominant genera among 80 endophytic isolates. (B) Salt tolerance phenotypes of wild soybean accessions, including the salt‐sensitive control line 17 (CK), and accessions 41 and 77. Two‐week‐old seedlings grown in sterilised vermiculite were carefully rinsed and transferred to MS liquid medium containing 120 mM NaCl. Phenotypes were documented following 4 days of salt treatment. Scale bar = 1 cm. (C) Growth of selected strains (77R1–77R6, 77S1–77S3, 77L1) under different NaCl concentrations (200, 300, 500 mM) and pH levels (7.0 or 9.0). ‘+++’, ‘++’, ‘+’, and ‘−’ indicate strong, moderate, weak, and no growth (the strain cannot survive). (D) Phenotypic comparison of soybean bud growth under 150 mM NaCl in control (CK) versus 77S3inoculated plants. Scale bar = 10 mm. (E) Quantification of bud length under the conditions shown in (D). (F) Statistical analyses of stem height in control and 77S3‐inoculated soybean seedlings under 150 mM NaCl. (E, F) Data represent mean ± SD (*n* = 5). Asterisks indicate significant differences from the control (*p < 0.05, two‐tailed *t*‐test).
**Figure S2:** Genomic features and MLSA‐based phylogenetic analysis of strain 77S3. (A) Schematic representation of the complete draft genome of strain 77S3, showing major genomic landmarks used for taxonomic classification and subsequent genomic analyses. (B) Genomic organisation of the six housekeeping genes (*dnaJ, dnaK, groEL, gyrA, recA, gyrB*) included in the multilocus sequence analysis (MLSA). Gene lengths and positions correspond to the whole‐genome assembly of strain 77S3 (GWH accession: GWHHAGN00000000.1). Concatenated sequences of these genes were used for phylogenetic reconstruction. Genomic classification via GTDB‐TK (v2.4.0; ANI estimation with skani) identified strain 77S3 as *Pseudomonas oryzihabitans* , with ANI = 98.33% and alignment fraction = 0.925 relative to the type strain (GTDB species threshold: ANI ≥ 95%, AF ≥ 0.5).
**Figure S3:** Transcriptome reprogramming analysis induced by 77S3 under control and salt‐stress conditions. (A) PCA analysis of transcriptomes from non‐inoculated and 77S3‐inoculated soybean roots under 0 mM or 150 mM NaCl conditions. Only transcripts with TPM > 1 were included. (B) Volcano plot of differentially expressed genes (DEGs) in soybean roots inoculated with *Pseudomonas* sp. 77S3 under salt‐stress versus control conditions. DEGs were identified using thresholds of |log_2_(fold change)| ≥ 1 and FDR < 0.05. The *x*‐axis represents log_2_(fold change), and the *y*‐axis shows –log_10_(*p*‐value). Significantly up‐regulated and down‐regulated genes are coloured red and blue, respectively; non‐significant genes are shown in grey. A pie chart adjacent to the plot summarises the proportions of up‐ and down‐regulated DEGs. (C‐D) Bubble plots of KEGG pathway enrichment analysis for DEGs: 77S3‐0 versus CK‐0 (C) and 77S3‐150 versus CK‐150 (D). The size of the bubble represents the number of genes, and the colour represents the value of −log10(*p*‐value). The *x*‐axis indicates the enrichment level, represented as the gene ratio, and the y‐axis displays the pathway (*q*‐value < 0.05).
**Figure S4:** Gene Ontology (GO) enrichment analysis of DEGs under salt stress with or without inoculation of *Pseudomonas* sp. 77S3. (A–C) Bar plots showing significantly enriched GO terms (*q*‐value < 0.05) for DEGs identified under salt stress in: shared responses regardless of inoculation status (A), non‐inoculated control plants (B), and *Pseudomonas* sp. 77S3‐inoculated plants (C). The *x*‐axis represents −log_10_(*p*‐value), and the *y*‐axis displays enriched functional terms.
**Figure S5:** Metabolic reprogramming and integrated multi‐omics analysis under salt stress. (A) Principal Component Analysis (PCA) of metabolomic profiles from non‐inoculated and *Pseudomonas* sp. 77S3‐inoculated soybean roots under 0 mM or 150 mM NaCl conditions. Axes represent the first (PC1) and second (PC2) principal components. (B) Orthogonal Partial Least Squares‐Discriminant Analysis (OPLS‐DA) score plots comparing metabolic profiles of non‐inoculated (CK) and 77S3‐inoculated plants at 0 and 150 mM NaCl. The predictive component (T score[1]) is shown on the x‐axis and the orthogonal component (Orthogonal T score[1]) on the *y*‐axis. (C) KEGG pathway enrichment analysis of DEMs for 77S3‐inoculated versus non‐inoculated plants under salt stress (150 mM NaCl). Bubble size indicates the number of metabolites mapped to each pathway; colour represents –log_10_(*p*‐value). The *x*‐axis shows the gene ratio, and the *y*‐axis lists significantly enriched pathways (*q*‐value < 0.05).
**Figure S6:** Effects of strain 77S3 on nitrogen allocation, nitrate transporter gene expression, and ion homeostasis under salt stress. (A) Detection of *nif*H gene. Lanes from left to right: DNA Marker, PCR product of *nif*H gene, ddH_2_O (negative control). (B) Relative transcript levels of nitrate transporter genes *NRT1.1*, *NRT2.4*, and *NRT3.1* in soybean roots inoculated with or without 77S3 under 0 and 150 mM NaCl conditions as determined by RT–qPCR. Transcript abundance was normalised to the endogenous reference gene *GmTUB2*. (C‐D) N concentration in stems and roots, and the stem‐to‐root N concentration ratio (stem/root) of control and 77S3‐inoculated soybean plants grown under 0 mM (C) and 150 mM (D) NaCl treatments. (E) K^+^ content in the roots of empty‐vector (EV) control and *nrt1.5* mutants under 0 and 150 mM salt conditions. (B–E) Data are presented as mean ± SD, with *n* = 3 for panels B, D and E and *n* = 5 for panel C. Significant differences were determined by two‐tailed Student's *t*‐test (*p < 0.05, **p < 0.01, ***p < 0.001; ns, not significant).

**Table S1:** Primers used in this study.
**Table S2:** Salinity tolerance of all endophytes.
**Table S3:** Summary of transcriptome sequencing data quality and alignment metrics.
**Table S4:** Number and list of genes expressed (TPM > 1) in soybean roots across experimental conditions.
**Table S5:** Differentially expressed genes (DEGs) in response to salt stress (CK_0 vs. CK_150) in non‐inoculated roots (|log_2_FC| > 1, FDR ≤ 0.05).
**Table S6:** DEGs in response to salt stress (77S3_0 vs. 77S3_150) in 77S3‐inoculated roots (|log_2_FC| > 1, FDR ≤ 0.05).
**Table S7:** KEGG pathway enrichment analysis of DEGs in non‐salt vs. salt‐treated roots, with or without inoculation of *Pseudomonas* sp. 77S3.
**Table S8:** Gene Ontology (GO) term enrichment analysis of DEGs in non‐salt vs. salt roots, with or without inoculation of *Pseudomonas* sp. 77S3.
**Table S9:** Differentially expressed metabolites (DEMs) between non‐inoculated and 77S3‐inoculated roots at 0 mM NaCl (|log_2_FC| > 1 or VIP > 1.0).
**Table S10:** DEMs between non‐inoculated and 77S3‐inoculated roots at 150 mM NaCl (|log_2_FC| > 1 or VIP > 1.0).
**Table S11:** KEGG enrichment analysis of DEMs in non‐inoculated and 77S3‐inoculated plants.
**Table S12:** Pearson correlations between DEGs and DEMs.

## Data Availability

All data supporting the findings of this study are included either within the article and its Supporting Information files or are available from the corresponding author upon reasonable request.
