# Supplementary material for: Reprogramming of Gene Transcripts and Metabolites by the Wild Soybean Endophyte Pseudomonas sp. 77S3 Improves Soybean Salt Tolerance
Source: Plant Biotechnol J. 2025 Dec 29;24(4):2704–21. doi: 10.1111/pbi.70514 (PMC13140724; doi:10.1111/pbi.70514)
Supplement: Supplementary file 1 — Figure S1: Identification of endophytic isolates and evaluation of their salt tolerance and plant growthpromoting effects. (A) Taxonomic distribution of the dominant genera among 80 endophytic isolates. (B) Salt tolerance phenotypes of wild soybean accessions, including the salt‐sensitive control line 17 (CK), and accessions 41 and 77. Two‐week‐old seedlings grown in sterilised vermiculite were carefully rinsed and transferred to MS liquid medium containing 120 mM NaCl. Phenotypes were documented following 4 days of salt treatment. Scale bar = 1 cm. (C) Growth of selected strains (77R1–77R6, 77S1–77S3, 77L1) under different NaCl concentrations (200, 300, 500 mM) and pH levels (7.0 or 9.0). ‘+++’, ‘++’, ‘+’, and ‘−’ indicate strong, moderate, weak, and no growth (the strain cannot survive). (D) Phenotypic comparison of soybean bud growth under 150 mM NaCl in control (CK) versus 77S3inoculated plants. Scale bar = 10 mm. (E) Quantification of bud length under the conditions shown in (D). (F) Statistical analyses of stem height in control and 77S3‐inoculated soybean seedlings under 150 mM NaCl. (E, F) Data represent mean ± SD (n = 5). Asterisks indicate significant differences from the control (*p < 0.05, two‐tailed t‐test). Figure S2: Genomic features and MLSA‐based phylogenetic analysis of strain 77S3. (A) Schematic representation of the complete draft genome of strain 77S3, showing major genomic landmarks used for taxonomic classification and subsequent genomic analyses. (B) Genomic organisation of the six housekeeping genes (dnaJ, dnaK, groEL, gyrA, recA, gyrB) included in the multilocus sequence analysis (MLSA). Gene lengths and positions correspond to the whole‐genome assembly of strain 77S3 (GWH accession: GWHHAGN00000000.1). Concatenated sequences of these genes were used for phylogenetic reconstruction. Genomic classification via GTDB‐TK (v2.4.0; ANI estimation with skani) identified strain 77S3 as Pseudomonas oryzihabitans , with ANI = 98.33% and alignment fra [file PBI-24-2704-s002.docx]

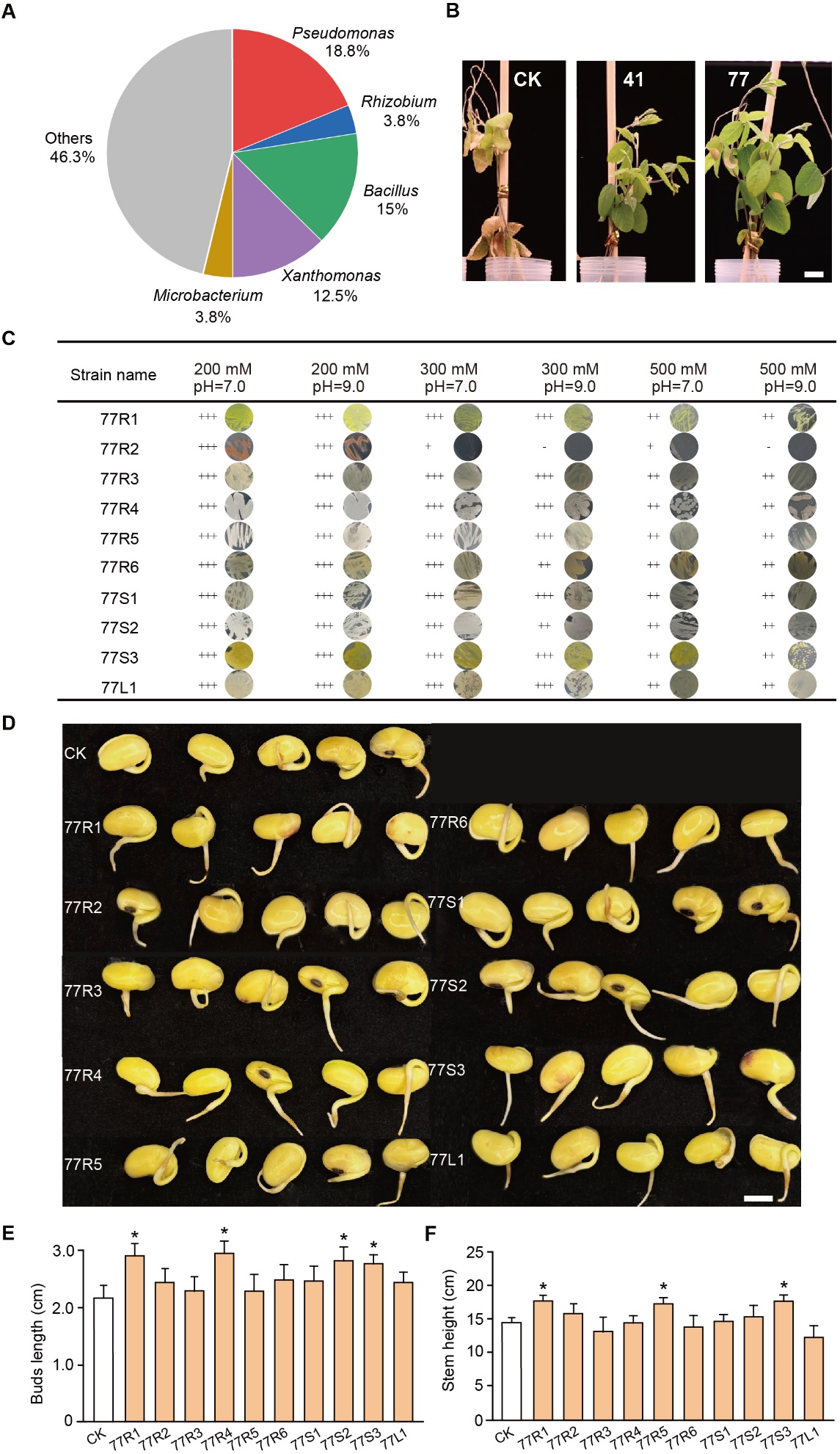


**Figure S1 Identification of endophytic isolates and evaluation of their salt tolerance and plant growth‑promoting effects.** **(A)** Taxonomic distribution of the dominant genera among 80 endophytic isolates. **(B)** Salt tolerance phenotypes of wild soybean accessions, including the salt-sensitive control line 17 (CK), and accessions 41 and 77. Two-week-old seedlings grown in sterilized vermiculite were carefully rinsed and transferred to MS liquid medium containing 120 mM NaCl. Phenotypes were documented following 4 days of salt treatment. Scale bar = 1cm. **(C)** Growth of selected strains (77R1–77R6, 77S1–77S3, 77L1) under different NaCl concentrations (200, 300, 500 mM) and pH levels (7.0 or 9.0). "+++", "++", "+", and "−" indicate strong, moderate, weak, and no growth (the strain cannot survive). **(D)** Phenotypic comparison of soybean bud growth under 150 mM NaCl in control (CK) versus 77S3‑inoculated plants. Scale bar = 10 mm. **(E)** Quantification of bud length under the conditions shown in (D). **(F)** Statistical analyses of stem height in control and 77S3‑inoculated soybean seedlings under 150 mM NaCl. **(E**, **F)** Data represent mean ± SD (n = 5). Asterisks indicate significant differences from the control (**p* < 0.05, two‑tailed t‑test).


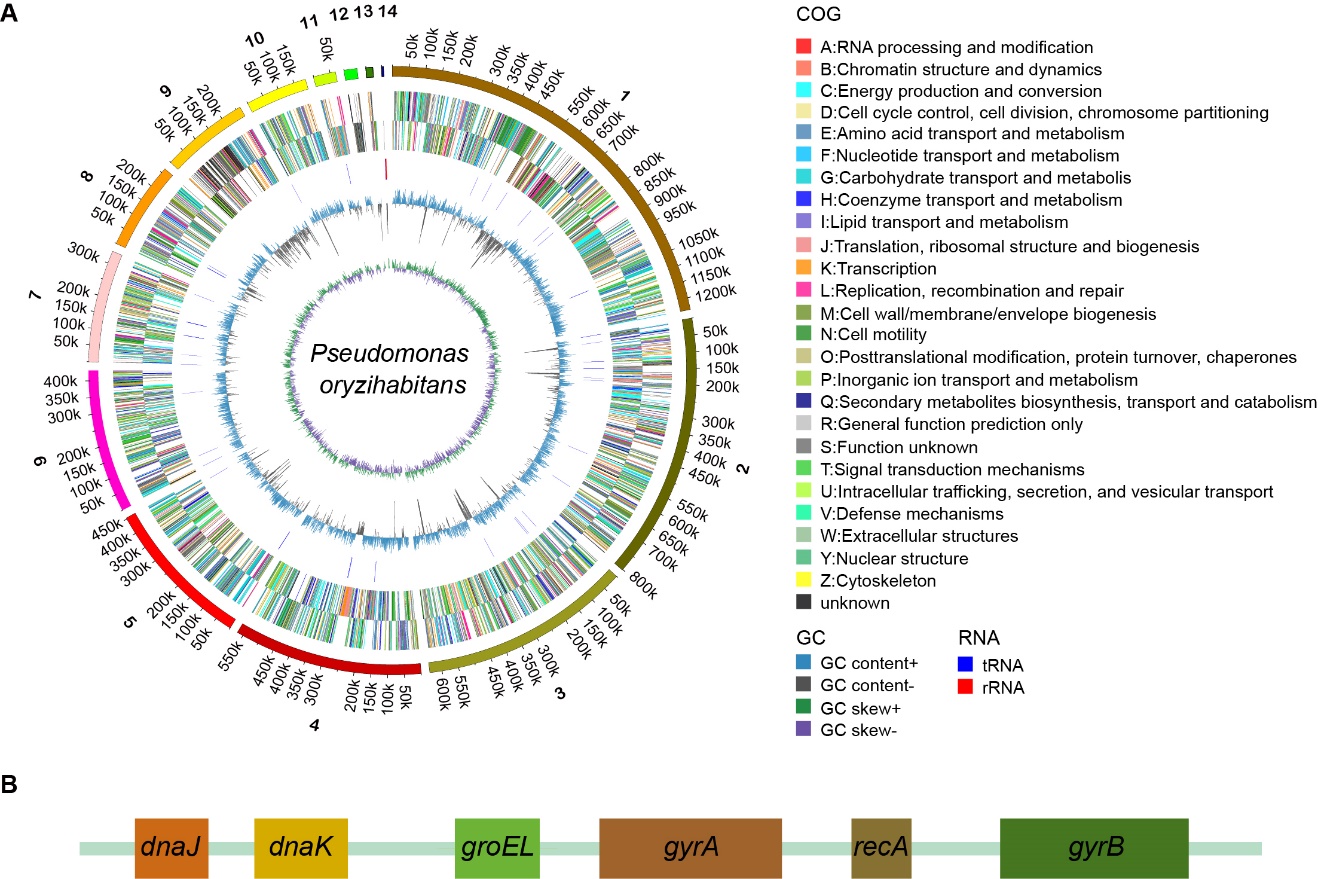


**Figure S2 Genomic features and MLSA-based phylogenetic analysis of strain 77S3.** **(A)** Schematic representation of the complete draft genome of strain 77S3, showing major genomic landmarks used for taxonomic classification and subsequent genomic analyses.
**(B)** Genomic organization of the six housekeeping genes (*dnaJ, dnaK, groEL, gyrA, recA, gyrB*) included in the multilocus sequence analysis (MLSA). Gene lengths and positions correspond to the whole-genome assembly of strain 77S3 (GWH accession: GWHHAGN00000000.1). Concatenated sequences of these genes were used for phylogenetic reconstruction. Genomic classification via GTDB-TK (v2.4.0; ANI estimation with skani) identified strain 77S3 as *Pseudomonas oryzihabitans*, with ANI = 98.33% and alignment fraction = 0.925 relative to the type strain (GTDB species threshold: ANI ≥ 95%, AF ≥ 0.5).


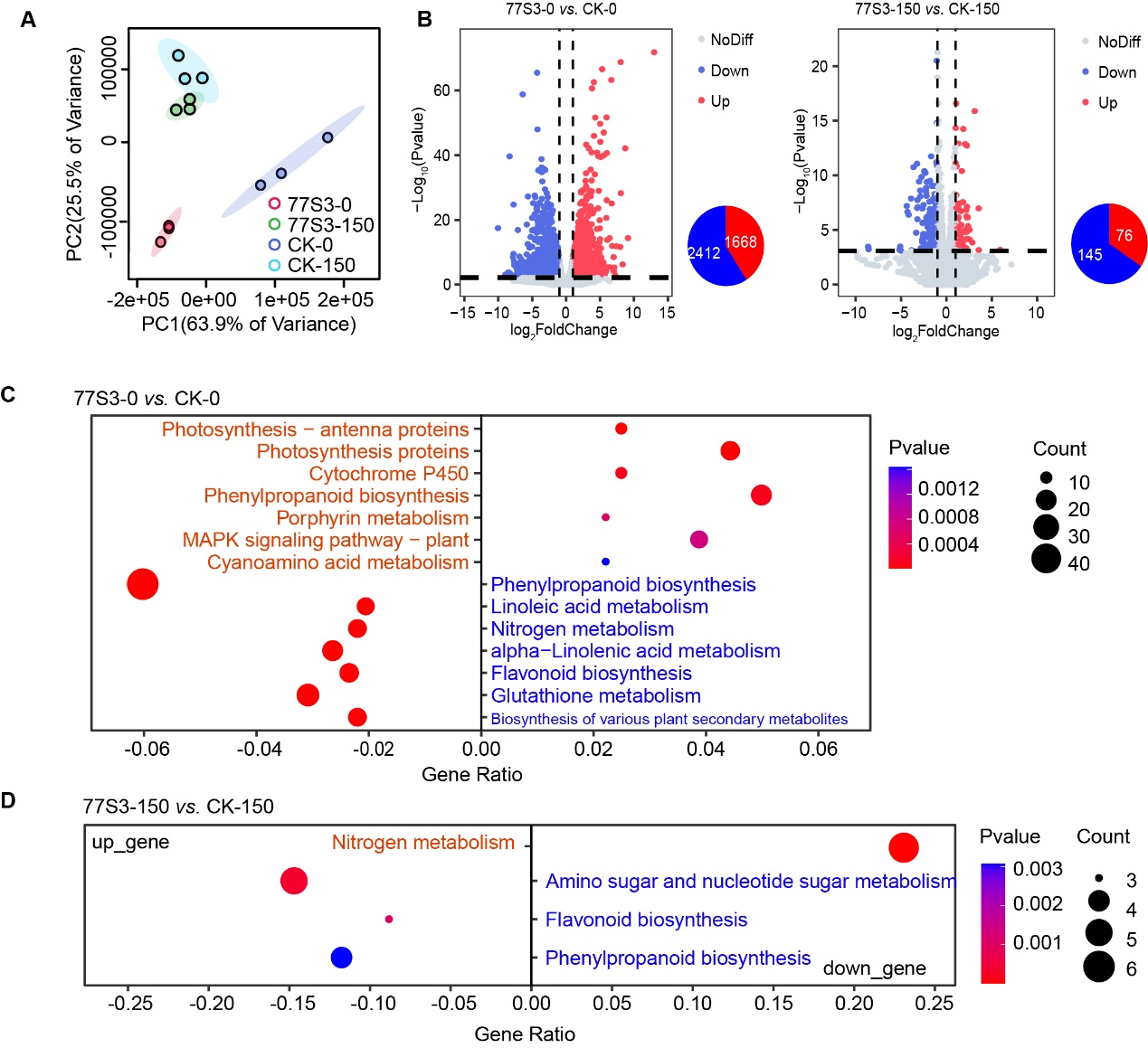


**Figure S3 Transcriptome reprogramming analysis induced by 77S3 under control and salt-stress conditions.** (**A**) of transcriptomes from non-inoculated and 77S3-inoculated soybean roots under 0 mM or 150 mM NaCl conditions. Only transcripts with TPM >1 were included. (**B**) Volcano plot of differentially expressed genes (DEGs) in soybean roots inoculated with *Pseudomonas* sp. 77S3 under salt-stress versus control conditions. DEGs were identified using thresholds of |log₂(fold change)| ≥ 1 and FDR < 0.05. The x-axis represents log₂(fold change), and the y-axis shows -log₁₀(p-value). Significantly upregulated and downregulated genes are colored red and blue, respectively; nonsignificant genes are shown in gray. A pie chart adjacent to the plot summarizes the proportions of up- and down-regulated DEGs. (**C-D**) Bubble plots of KEGG pathway enrichment analysis for DEGs: 77S3-0 versus CK-0 (**C**) and 77S3-150 versus CK-150 (**D**). The size of the bubble represents the number of genes, and the color represents the value of -log10(pvalue). The x-axis indicates the enrichment level, represented as the gene ratio, and the y-axis displays the pathway (*q*-value < 0.05).


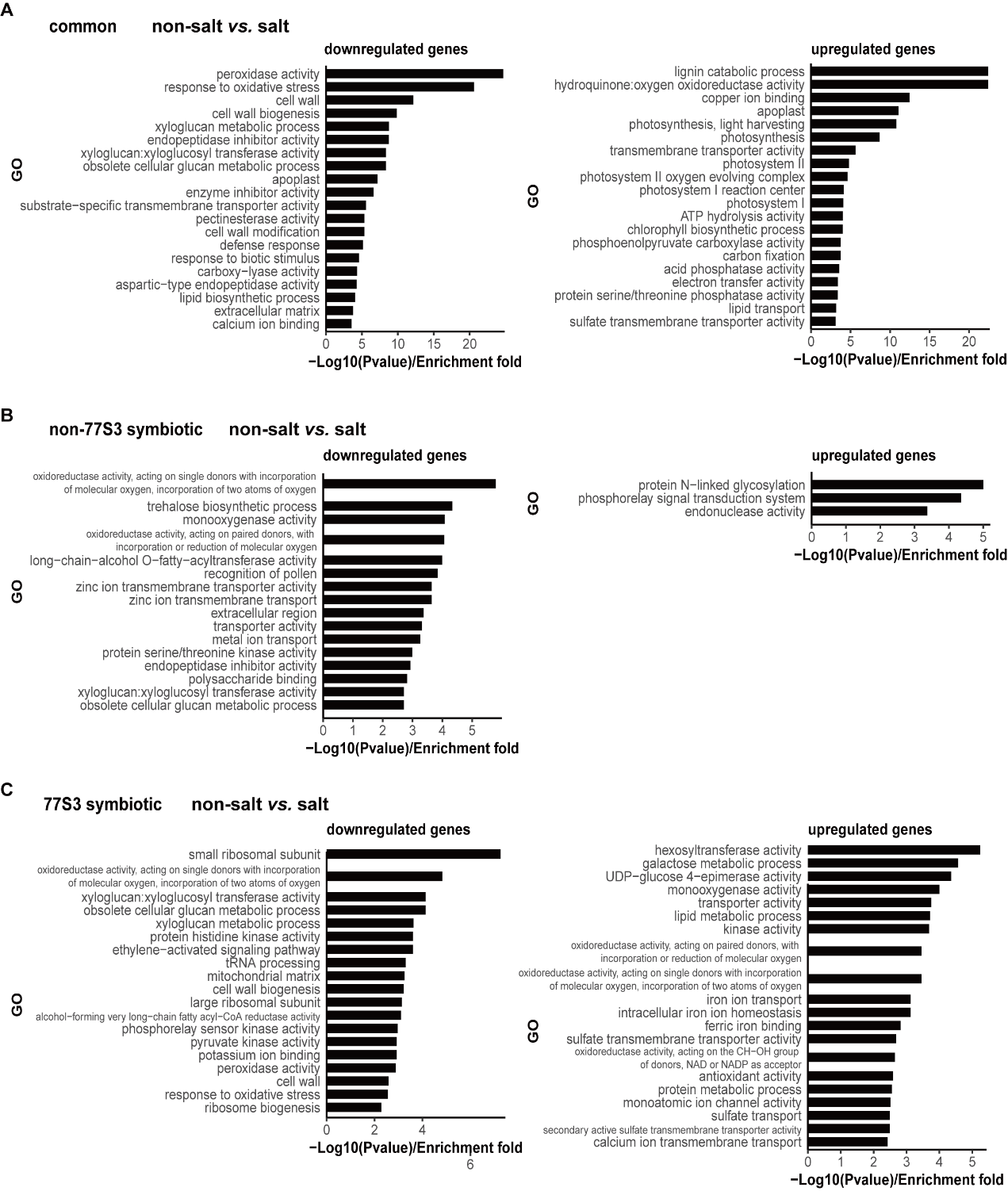
**Figure S4 Gene Ontology (GO) enrichment analysis of DEGs under salt stress with or without inoculation of *Pseudomonas* sp. 77S3. (A–C)** Bar plots showing significantly enriched GO terms (*q*-value < 0.05) for DEGs identified under salt stress in: shared responses regardless of inoculation status (**A**), non-inoculated control plants (**B**), and *Pseudomonas* sp. 77S3-inoculated plants (C). The x-axis represents –log₁₀(p-value), and the y-axis displays enriched functional terms.


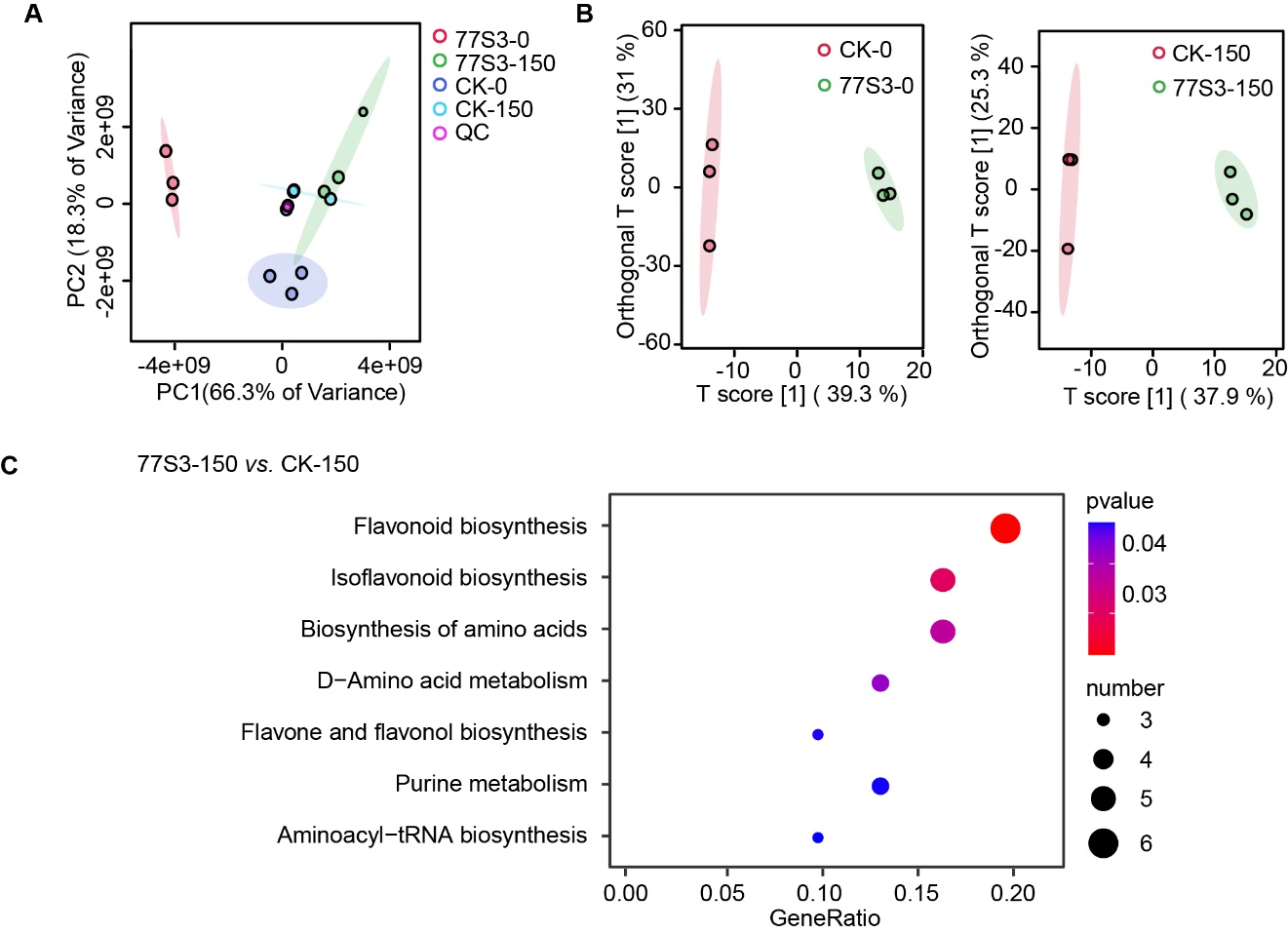


**Figure S5 Metabolic reprogramming and integrated multi-omics analysis under salt stress.** **(A)** Principal Component Analysis (PCA) of metabolomic profiles from non-inoculated and *Pseudomonas* sp. 77S3-inoculated soybean roots under 0 mM or 150 mM NaCl conditions. Axes represent the first (PC1) and second (PC2) principal components. **(B)** Orthogonal Partial Least Squares-Discriminant Analysis (OPLS-DA) score plots comparing metabolic profiles of non-inoculated (CK) and 77S3-inoculated plants at 0 mM and 150 mM NaCl. The predictive component (T score[1]) is shown on the x-axis and the orthogonal component (Orthogonal T score[1]) on the y-axis. **(C)** KEGG pathway enrichment analysis of DEMs) for 77S3-inoculated versus non-inoculated plants under salt stress (150 mM NaCl). Bubble size indicates the number of metabolites mapped to each pathway; color represents –log₁₀(*p*-value). The x-axis shows the gene ratio, and the y-axis lists significantly enriched pathways (*q*-value < 0.05).


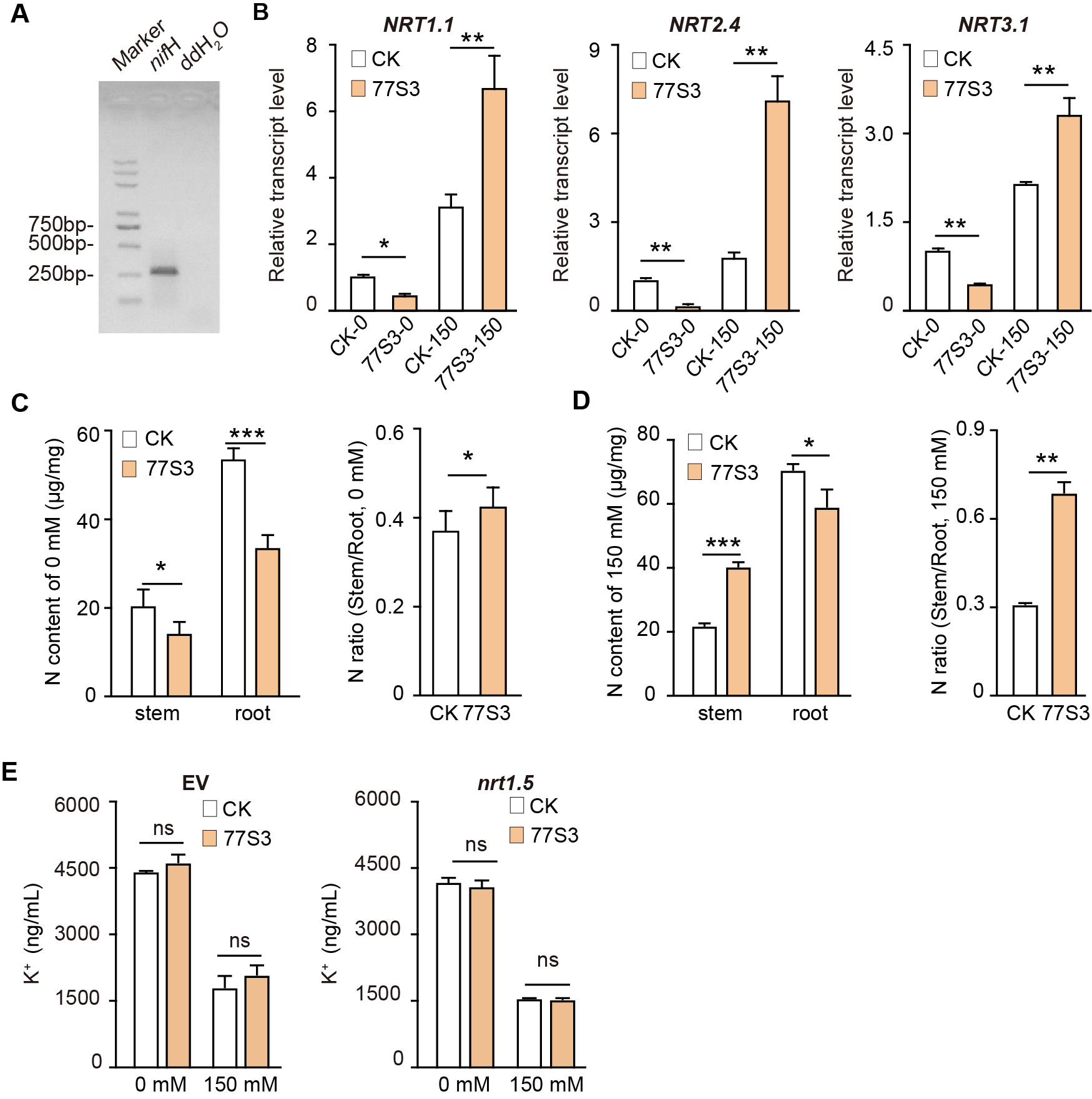


**Figure S6** **Effects of strain 77S3 on nitrogen allocation, nitrate transporter gene expression, and ion homeostasis under salt stress. (A)** Detection of *nif*H gene. Lanes from left to right: DNA Marker, PCR product of *nifH* gene, ddH₂O (negative control). (**B**) Relative transcript levels of nitrate transporter genes *NRT1.1*, *NRT2.4*, and *NRT3.1* in soybean roots inoculated with or without 77S3 under 0 mM and 150 mM NaCl conditions. as determined by RT‒qPCR. Transcript abundance was normalized to the endogenous reference gene *GmTUB2*. (**C-D**) N concentration in stems and roots, and the stem-to-root N concentration ratio (stem/root) of control and 77S3-inoculated soybean plants grown under 0 mM (C) and 150 mM (D) NaCl treatments. (**E**) K⁺ content in the roots of empty‑vector (EV) control and *nrt1.5* mutants under 0 mM and 150 mM salt conditions. (**B–E**) Data are presented as mean ± SD, with n = 3 for panels B, D–E and n = 5 for panel C. Significant differences were determined by two-tailed Student's t-test (**p* < 0.05, ***p* < 0.01, ****p* < 0.001; ns, not significant).

**Table S1** Primers used in this study.

**Table S2** Salinity tolerance of all endophytes.

**Table S3** Summary of transcriptome sequencing data quality and alignment metrics.

**Table S4** Number and list of genes expressed (TPM > 1) in soybean roots across experimental conditions.

**Table S5** Differentially expressed genes (DEGs) in response to salt stress (CK_0 vs. CK_150) in non-inoculated roots (│log₂FC│> 1, FDR ≤ 0.05).

**Table S6** DEGs in response to salt stress (77S3_0 vs. 77S3_150) in 77S3-inoculated roots (│log₂FC│> 1, FDR ≤ 0.05).

**Table S7** KEGG pathway enrichment analysis of DEGs in non-salt vs. salt-treated roots, with or without inoculation of *Pseudomonas* sp. 77S3.

**Table S8** Gene Ontology (GO) term enrichment analysis of DEGs in non-salt vs. salt roots, with or without inoculation of *Pseudomonas* sp. 77S3.

**Table S9** Differentially expressed metabolites (DEMs) between non-inoculated and 77S3-inoculated roots at 0 mM NaCl (│log₂FC│> 1 or VIP > 1.0).

**Table S10** DEMs between non-inoculated and 77S3-inoculated roots at 150 mM NaCl (│log₂FC│> 1 or VIP > 1.0).

**Table S11** KEGG enrichment analysis of DEMs in non-inoculated and 77S3-inoculated plants.

**Table S12** Pearson correlations between DEGs and DEMs.
